# Metabolomic profiling of bovine leucocytes transformed by *Theileria annulata* under BW720c treatment

**DOI:** 10.1186/s13071-022-05450-0

**Published:** 2022-10-05

**Authors:** Hong-xi Zhao, Xia Li, Jun-long Liu, Gui-quan Guan, Xin-gang Dan

**Affiliations:** 1 School of Agriculture, Ningxia University, Yinchuan, 750021 People’s Republic of China; 2 State Key Laboratory of Veterinary Etiological Biology, Key Laboratory of Veterinary Parasitology of Gansu Province, Lanzhou Veterinary Research Institute, Chinese Academy of Agricultural Sciences, Xujiaping 1, Lanzhou, 730046 People’s Republic of China

**Keywords:** *Theileria annulata*, Metabolomics, Pathway, Buparvaquone (BW720c)

## Abstract

**Background:**

When *Theileria annulata* infects host cells, it undertakes unlimited proliferation as tumor cells. Although the transformed cells will recover their limited reproductive characteristics and enter the apoptosis process after treatment with buparvaquone (BW720c), the metabolites and metabolic pathways involved are not clear.

**Methods:**

The transformed cells of *T. annulata* were used as experimental materials, and the buparvaquone treatment group and DMSO control group were used. Qualitative and quantitative analysis was undertaken of 36 cell samples based on the LC–QTOF platform in positive and negative ion modes. The metabolites of the cell samples after 72 h of drug treatment were analyzed, as were the different metabolites and metabolic pathways involved in the BW720c treatment. Finally, the differential metabolites and metabolic pathways in the transformed cells were found.

**Results:**

A total of 1425 metabolites were detected in the negative ion mode and 1298 metabolites were detected in the positive ion mode. After drug treatment for 24 h, 48 h, and 72 h, there were 56, 162, and 243 differential metabolites in negative ion mode, and 35, 121, and 177 differential metabolites in positive ion mode, respectively. These differential metabolites are mainly concentrated on various essential amino acids.

**Conclusion:**

BW720c treatment induces metabolic disturbances in *T. annulata*-infected cells by regulating the metabolism of leucine, arginine, and l-carnitine, and induces host cell apoptosis.

**Graphical abstract:**

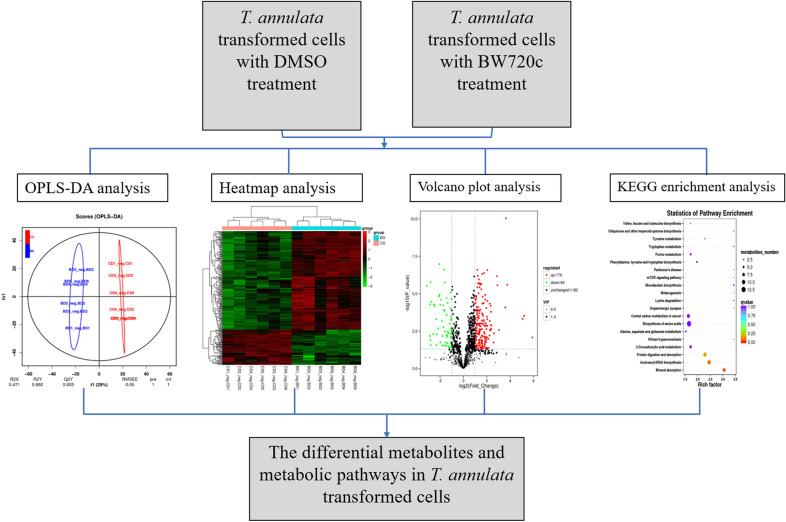

**Supplementary Information:**

The online version contains supplementary material available at 10.1186/s13071-022-05450-0.

## Background

*Theileria annulata* is one of the main pathogens which are harmful to cattle, causing infected animals to show high fever, jaundice, hemoglobinuria, anemia, and swollen lymph nodes on the body surface [1–5]. *Theileria annulata* is an obligate intracellular protozoan parasite, which can induce bovine tropical theileriosis [6]. It is estimated that approximately 250 million cattle globally are at risk of tropical theileriosis, which has caused large economic losses to the cattle industry [7]. *Theileria annulata* has a complex life cycle composed of two hosts. Firstly, after completing the sexual reproduction stage inside the intestine of ticks, *T. annulata* migrates to the acinar cells of the tick’s salivary glands, where it matures into sporozoites and is released in the saliva [8]. After entering the bovine bloodstream, the sporozoite invades monocytes, macrophages, and B lymphocytes. It is then transformed into the schizont of *T. annulata* and changes several signaling pathways, which ultimately leads to host cell transformation [9–11].

When cells were cultured in vitro, it was found that the transformation of lymphocytes caused by the *T. annulata* schizont could be reversed by the anti-parasite drug buparvaquone, whereupon the transformed cells returned to their limited reproduction and entered the normal apoptosis process. However, this drug had no effect on normal cells [12]. Research on this transformation mechanism is not only important for the prevention and control of *T. annulata* [13, 14], but also of great significance for revealing the mechanism of cancer in humans [15]. The mechanisms of the metabolites and metabolic pathways are not clear. Recent developments in metabolomics have been widely used in drug research and development, molecular physiology and pathology, nutrition, environmental science, and other fields [16]. However, the metabolomics of host cells under parasite influence has only recently attracted attention [17].

Metabolomics deals with substances with molecular weight less than 1000 Da, including lipids, carbohydrates, amino acids, nucleotides, and peptides. [18–20]. It encompasses the large-scale research on the metabolism of cells, and has been investigated for more than 20 years [21, 22]. Metabolomics is now a commonly used experimental system biology tool, with practical application in plant, microbe, and mammal research [23–25]. The study of metabolomics can be divided into targeted metabolomics and non-targeted metabolomics [20]. Non-targeted metabolomics aims to identify all metabolites in a sample from the perspective of global analysis. The advantage of this model is to determine different metabolites comprehensively, which can be used for the preliminary screening of diagnostic markers [26]. Therefore, it was selected as the analysis method in this study. The objects of metabolomic detection can be tissues, cells, plasma, urine, and so on [27]. Cell metabolomics mainly focuses on the changes in complex intracellular and extracellular metabolites, in which this study uses the infected cells as the model. The transformed cells were studied for changes in differential metabolites and metabolic pathways under treatment, revealing the effect of *T. annulata* infection on the metabolome of bovine host cells and clarifying the mechanism that *T. annulata* induces in host cell transformation, as well as providing a theoretical basis for the prevention of *T. annulata* infection. Metabolic pathways can be used to identify the pathways of differential metabolite action. The analysis of these metabolic and regulatory pathways can provide a more comprehensive and systematic understanding of biological process changes incurred by both the parasites and the pathogenesis of disease traits. This study aimed to lay a foundation for understanding the interaction between *T. annulata* and host cells and reveal its transformation mechanism.

## Methods

### Cell culture

A 17-generation schizont-infected bovine lymphocyte line of *T. annulata* was provided by the Lanzhou Veterinary Research Institute Platform-TDRC-22, Chinese Academy of Agricultural Sciences. Infected lymphocytes were placed in water at 37 °C and centrifuged at 1000 r/min for 5 min. The pellet was then transferred into RPMI-1640 with 10% fetal bovine serum and 16 ng/μl gentamicin in a culture flask and kept in a 37 °C incubator containing 5% CO_2_. For cell treatment, the cultured *T. annulata*-transformed cells were used as the research object, the BW720c (drug) cell treatment group was used as the test group, and the DMSO cell treatment group was used as the negative control group, with six replicates in each group. The cell concentration was set to 1 × 10^5^ cells/ml, the treatment group was added to 200 ng/ml BW720c drug using DMSO as solvent, and the same volume of DMSO was added to the control group. The cells were returned to the incubator for further culturing. Cells were counted at 0 h, 24 h, 48 h, and 72 h to ensure that the number of cells reached 1 × 10^7^ cells (the difference in the cell number is controlled within 1.2 times). The cell suspension was then removed from the culture flask and put into a 50 ml centrifuge tube and centrifuged at 620*g* at 4 °C for 5 min to remove the supernatant. The pellet was washed in 1 ml of phosphate-buffered saline (PBS) to remove residual culture fluid components, mixed, and centrifuged again at 620*g* at 4 °C for 3 min. This process was repeated three times. The cell pellets were then placed immediately in liquid nitrogen for 24 h and then stored at −80 °C.

### Sample preparation

A total of 1 × 10^7^ cells were transferred to a 1.5 ml Eppendorf (EP) tube, 300 μl of methanol and 20 μl of internal standard were added, and the mixture was vortexed for 30 s. The sample was subjected to ultrasonication for 10 min in an ice water bath to release metabolites by breaking the cells; then the sample was further incubated at −20 °C for 1 h. The metabolites were separated by centrifugation at 10,000×*g* for 15 min at 4 °C; the supernatant (200 μl) was removed and put into a 2 ml sample bottle; 20 μl of the sample was removed and mixed into the quality control (QC) sample. Online testing was undertaken with 200 μl.

### LC–QTOF-MS conditions

The detailed conditions of the liquid chromatography–quadrupole time-of-flight–mass spectrometry (LC–QTOF-MS) test are given in Additional file 1.

### Metabolomic data processing

The ionization source of the LC–QTOF-MS platform was electrospray ionization, with two ionization modes: positive (pos) and negative (neg). The two combinations provided a higher coverage rate and so made for better detection. The two groups of data were analyzed separately. Multivariate statistical analysis was used for the metabolomic data, comprising a principal component analysis (PCA) and orthogonal partial least squares discriminant analysis (OPLS-DA). Through OPLS-DA, the orthogonal variables which were not related to the classification variables were filtered out and the non-orthogonal and the orthogonal variables analyzed to obtain a more reliable correlation between the differences in metabolites and the experimental group. In this experiment, the OPLS-DA model was calculated using the R (3.3.2) package ropls. The reliability of the OPLS-DA model was verified. The screening criteria were fold change (FC) > 2, *P* < 0.05, and variable importance in projection (VIP) > 1. The heat map provided an intuitive visualization of the concentration of metabolites in the entire sample. The difference between *R*^2^ and *Q*^2^ decreased with the robustness of the score map.

### Functional analysis of the differential metabolite KEGG

KEGG (Kyoto Encyclopedia of Genes and Genomes) is a large database that integrates genome, chemistry, and system function information. KEGG PATHWAY is a set of manually drawn path diagrams to promote scholars' understanding of the interactions, reactions, and relationship networks of metabolic small molecules [28, 29]. Complex metabolic reactions are undertaken by different genes and proteins via complex pathways and networks, leading to systematic changes in metabolism, and aiding the determination of the pathway of differential metabolite action.

## Results

### Principal component analysis of the experimental group and control group

**Fig. 1:**
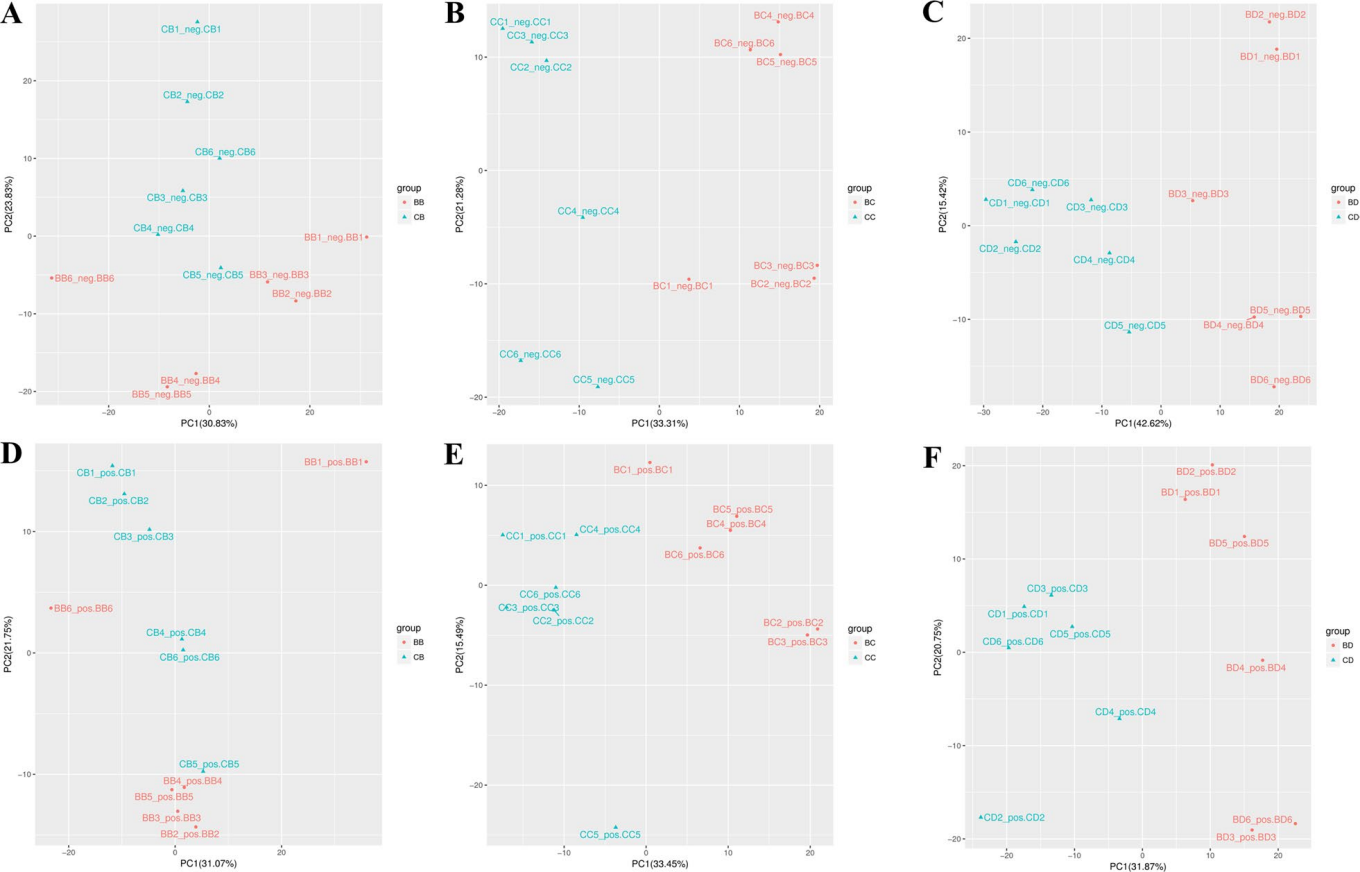
PCA scatter plots of the buparvaquone group and DMSO group. **a**–**c** Drug treatment at 24 h, 48 h, and 72 h, respectively (neg). **d**–**f** Drug treatment at 24 h, 48 h, and 72 h, respectively (pos). The *x*-axis represents PC1, the *y*-axis represents PC2. Symbols: BB, BC, and BD represent 24 h, 48 h, and 72 h of BW720c treatment, respectively; CB, CC, and CD represent 24 h, 48 h, and 72 h of DMSO treatment, respectively. The numbers 1–6 at the end of each string represent the samples in each group

The observation of the quality control results of the ion chromatography showed that the total ion chromatogram (TIC) peak retention times and peak areas of the six quality control samples overlapped well, which indicated that the samples were of good analytical quality and could be used for subsequent analysis (Additional file 1: Figure S1). On this basis, a dimensionality reduction analysis was performed on the data obtained by performing the PCA of the samples in neg and pos mode to gain a preliminary understanding of overall metabolic differences and intragroup variability. PCA was performed on metabolic data to identify global omics test result quality. As shown in Additional file 1: Figure S2, in both the neg mode and pos mode PCA plot, each group can be effectively separated. Further analysis of the test data of each group revealed that the differential groups in this experiment, according to the PCA (Fig. 1), were all evident at 24 h, 48 h, and 72 h with buparvaquone treatment, whether in neg or pos mode. As the processing time continued, the differential groups became more evident. The sample’s cluster at 48 h and 72 h was stronger than at 24 h, and there was no significant outlier in the experimental group (Fig. 1b, c, e, and f). A single discrete value was an anomaly in the sample handling process (Fig. 1d and e). It can be seen from Fig. 1 that the PCA scatter plot could only find the differences within the group.

### OPLS-DA of the experimental group and control group

**Fig. 2:**
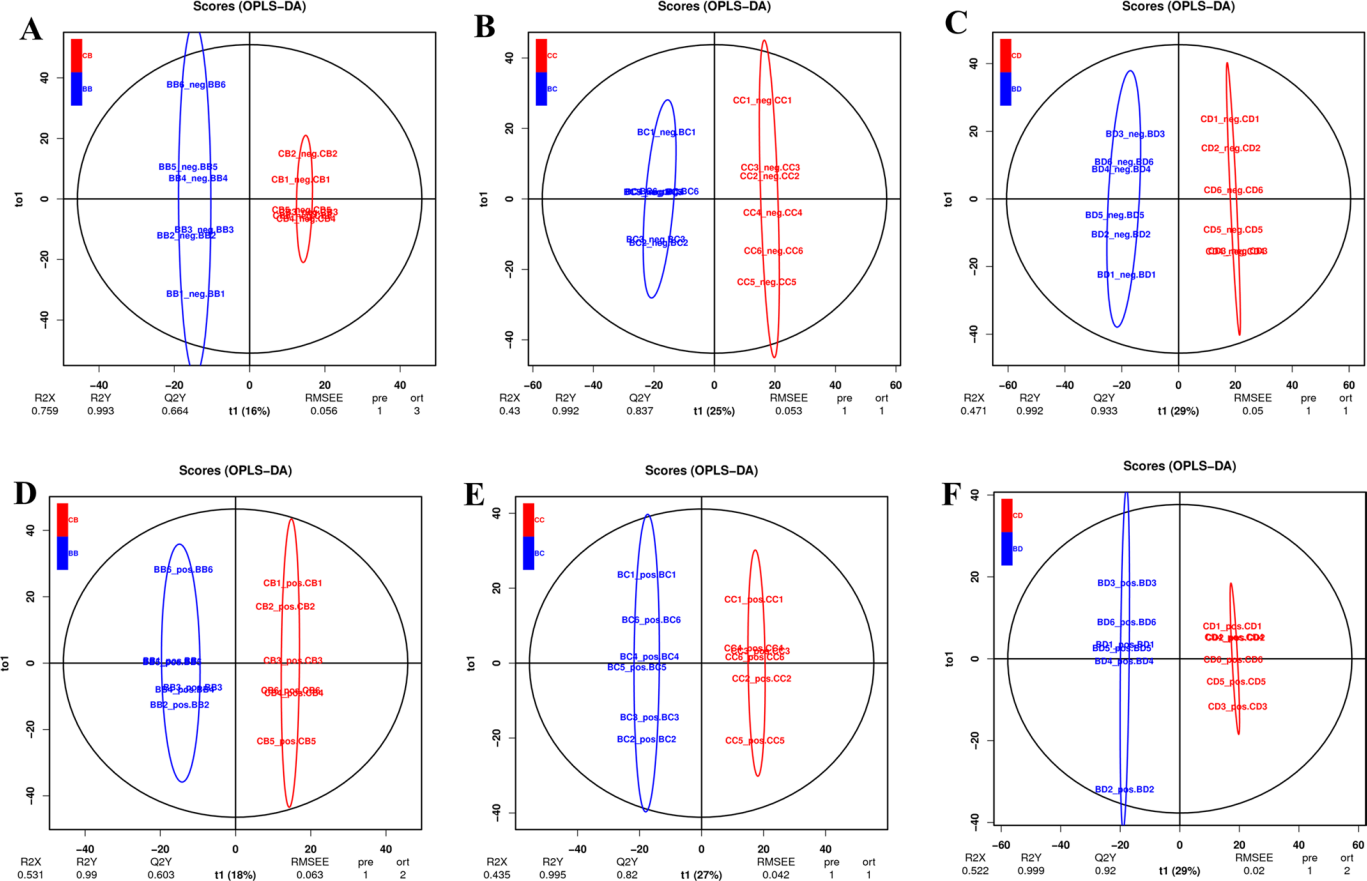
OPLS-DA scoring diagrams of the buparvaquone group and DMSO group. **a**–**c** 24 h, 48 h, and 72 h (neg); **d**–**f** 24 h, 48 h, and 72 h (pos). 24 h (*R*^2^*X* = 0.759, *R*^2^*Y* = 0.993, *Q*^2^ = 0.664 [neg]; *R*^2^*X* = 0.531, *R*^2^*Y* = 0.99, *Q*^2^ = 0.603 [pos]). 48 h (*R*^2^*X* = 0.43, *R*^2^*Y* = 0.992, *Q*^2^ = 0.837 [neg]; *R*^2^*X* = 0.435, *R*^2^*Y* = 0.995, *Q*^2^ = 0.82 ([pos]). 72 h (*R*^2^*X* = 0.471, *R*^2^*Y* = 0.992, *Q*^2^ = 0.933 [neg]; *R*^2^*X* = 0.552, *R*^2^*Y* = 0.999, *Q*^2^ = 0.92 [pos]). The *x*-axis represents t1, y-axis represents to 1. Symbol system: BB, BC, and BD represent 24 h, 48 h, and 72 h of BW720c treatment, respectively; CB, CC, and CD represent 24 h, 48 h, and 72 h of DMSO treatment, respectively. The numbers 1–6 at the end of the string are the number of samples in each group

The differences between the experimental group and the control group can be better explained by the OPLS-DA scoring diagram, with the analysis filtering out the irrelevant orthogonal signals to maximize differences. More reliable differential metabolites can be identified through this model. As shown in Fig. 2, clustering occurs among different groups in the model. The prediction parameters of the model are *R*^2^*X*, *R*^2^*Y*, and *Q*^2^, where *R*^2^*X* and *R*^2^*Y* represent the interpretation rate of the established model to the *X* and *Y* matrix, respectively, and *Q*^2^ represents the prediction ability of the model: the closer these three indices are to 1, the more stable and reliable the model. When *Q*^2^ > 0.5, it can be considered an effective model, and at *Q*^2^ > 0.9, it is considered an excellent model [30]. From Fig. 2 it can be seen that all models were reliable, which ensured the accuracy of the analysis results. In addition, a comparison of the *Q*^2^ value (Table 1) shows that the neg mode is better than the pos mode; the reliability and stability of the model constructed at 72 h are the most robust. This indicates that the difference in metabolites increased from the beginning of treatment to 72 h after BW720c treatment and then tended to stabilize. At this time, the analysis of the differences in metabolites and differential functional pathways was more reliable.

**Table 1:** Statistics of *Q*^2^ value of OPLS-DA under BW720c and control (DMSO) treatments

	*Q*^2^-value		
	24 h	48 h	72 h
neg	0.664	0.837	0.933
pos	0.603	0.820	0.920

**Fig. 3:**
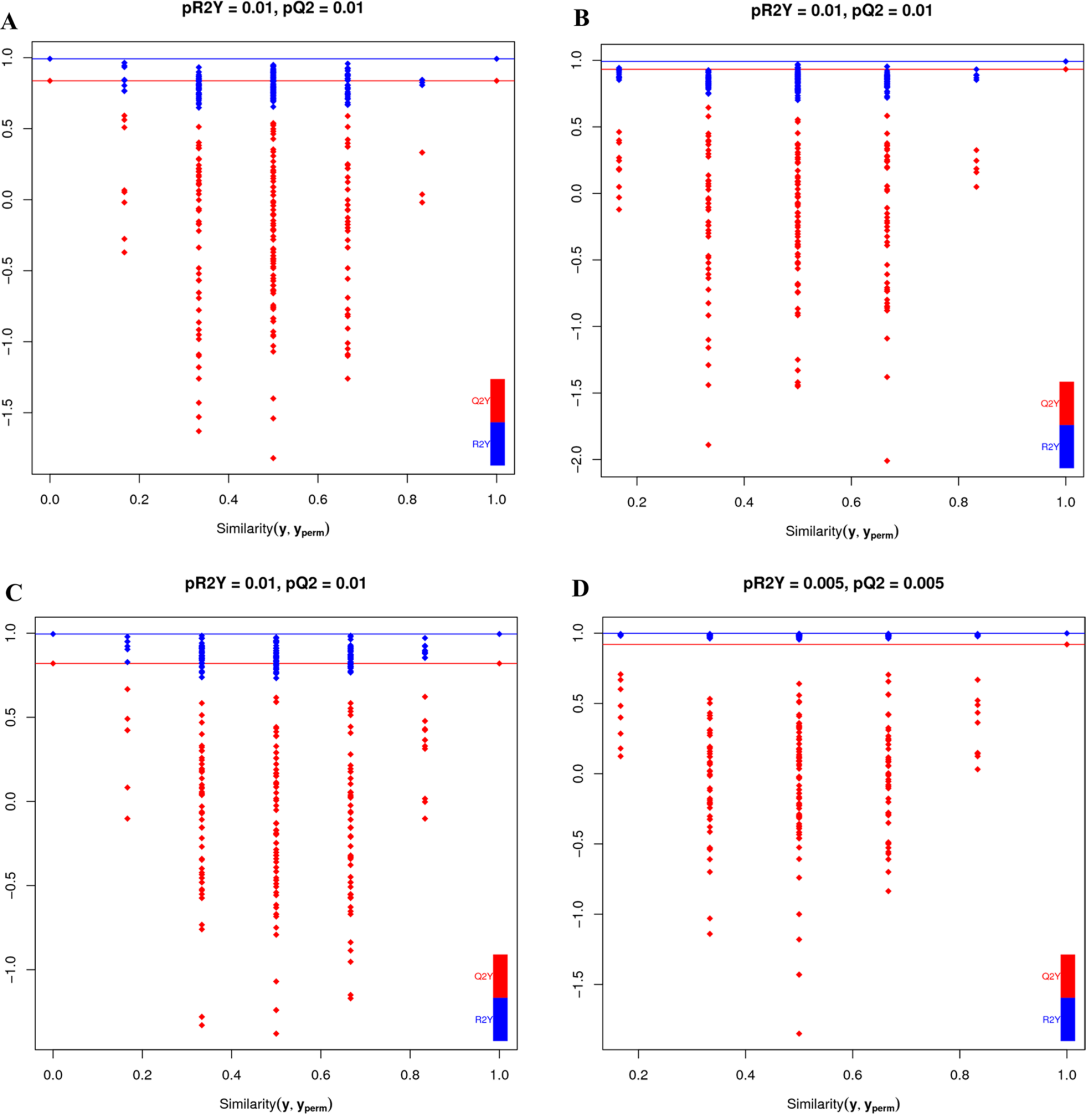
Permutation tests of the OPLS-DA model. **a**, **b** 48 h and 72 h (neg); **c**, **d** 48 h and 72 h (pos). 48 h (*pR*^2^*Y* = 0.01, *pQ*^2^ = 0.01 (neg); *pR*^2^*Y* = 0.01, *pQ*^2^ = 0.01 [pos]); 72 h (*pR*^2^*Y* = 0.01, *pQ*^2^ = 0.01 (neg); *pR*^2^*Y* = 0.005, *pQ*^2^ = 0.005 [pos])

The permutation test results are shown in Fig. 3. The horizontal line corresponds to *R*^2^ and *Q*^2^ of the original model, and the blue and red dots represent *R*^2^′ and *Q*^2^′ of the model after *y* substitution, respectively. If *R*^2^′ and *Q*^2^′ are smaller than *R*^2^ and *Q*^2^ of the original model, i.e., if the corresponding points do not exceed the corresponding lines, then the model is meaningful. It can be seen from Fig. 3 that the corresponding points do not exceed the corresponding lines, which shows that the OPLS-DA model is reliable.

### Screening of differential metabolites

**Fig. 4:**
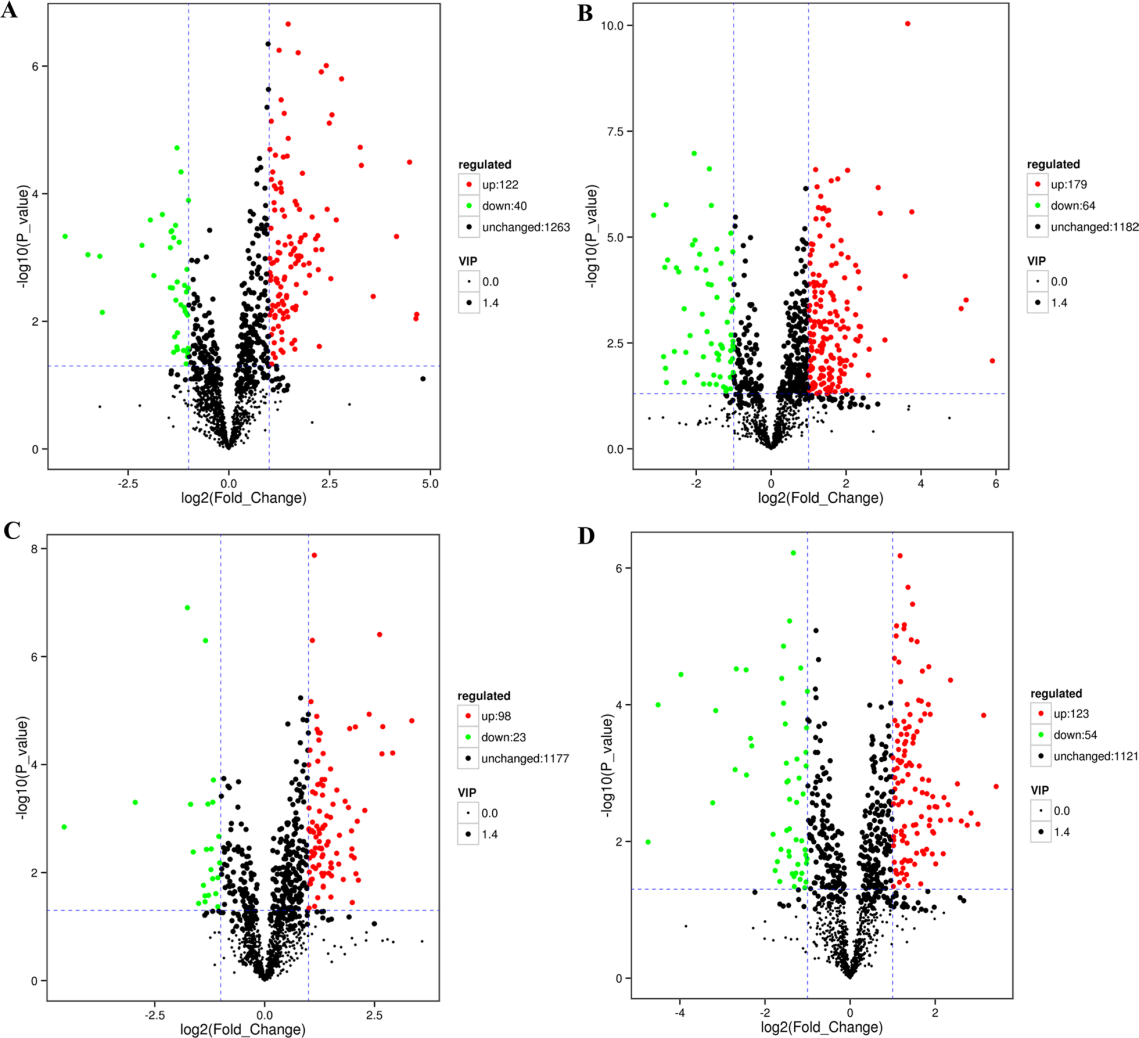
Metabolite differences in buparvaquone and DMSO treatments. **a**, **b** 48 h and 72 h (neg); **c**, **d** 48 h and 72 h (pos). The green dots represent the downregulated differential expression metabolites, the red dots represent the upregulated differential expression metabolites, and the black dots represent detected but nonsignificant metabolites. In neg ion mode, 2850 metabolites were found and 2596 metabolites were found in pos ion mode

In neg ion mode, there were 162 and 243 differential metabolites at 48 h and 72 h treatment, respectively, and in pos ion mode, 121 and 177 differential metabolites. Table 2 shows common differential metabolites in the two modes at 48 h and 72 h. According to the volcano plot, the difference in the expression level of metabolites in the two groups can be seen, as well as the statistical significance of that difference (Fig. 4). Each point in the volcano plot represents a metabolite, the abscissa represents the multiple change of each substance (log_2_), the ordinate represents the *P*-value (log_10_) of Student’s *t*-test, and the scatter size represents the VIP value of the OPLS-DA model. The larger the scatter, the greater the VIP value, and the more reliable the differential expression metabolite. The green dots represent the downregulated differential expression metabolites, the red dots represent the upregulated differential expression metabolites, and the black dots represent detected but nonsignificant metabolites. In Fig. 4, it can be seen that with the increase in processing time, the number of differential metabolites also increased, and there were more upregulated metabolites than downregulated ones.

**Table 2:** Common differential metabolites at 48 h and 72 h in both modes

Model	ID	Metabolite	Regulated	KEGG annotation
neg	meta_3	Dimethylformamide	Up	0
	meta_17	l-Serine	Up	15
	meta_22	Hypotaurine	Up	2
	meta_34	l-Threonine	Up	10
	meta_36	Tyramine	Up	3
	meta_38	Purine	Up	0
	meta_41	Picolinic acid	Down	2
	meta_51	l-Leucine	Up	11
	meta_56	d-Aspartic acid	Up	1
	meta_78	l-Glutamine	Up	17
	meta_99	Tryptamine	Up	3
	meta_102	l-Carnitine	Down	1
	meta_109	l-Phenylalanine	Up	10
	meta_120	Pro-Gly	Up	0
	meta_133	l-Tyrosine	Up	19
	meta_146	DL-Indole-3-lactic acid	Up	0
	meta_181	l-Tryptophan	Up	12
	meta_186	Methylthiouracil	Up	0
	meta_251	Meteloidine	Down	0
	meta_252	Tyrosyl-Glycine	Up	0
	meta_253	Tyr-Gly	Up	0
	meta_261	l-2-Amino-3-(oxalylamino)propanoic acid	Up	0
	meta_271	Pro-Glu	Up	0
	meta_275	2-Methylbutyroylcarnitine	Down	0
	meta_280	2,6-Diamino-4-hydroxy-5-*N*-methylformamidopyrimidine	Down	0
	meta_319	1-Amino-3-hydroxymethyl-5-methyl-adamantane	Up	0
	meta_324	Primaquine	Up	0
	meta_343	1-Chloro-2,2-bis(4′-chlorophenyl)ethylene	Up	0
	meta_425	Phe-Glu	Up	0
	meta_459	Xanthosine	Down	3
	meta_475	Flusilazole	Down	0
	meta_481	Arg-Thr	Up	0
	meta_502	Norelgestromin	Up	0
	meta_503	Oxprenolol	Up	0
	meta_520	Triflupromazine	Up	0
	meta_543	Adenosine monophosphate	Down	0
	meta_545	Butote	Up	0
	meta_589	4,7,10,13,1 6,19-Docosahexaenoic acid	Up	0
	meta_597	1,2-*O*-Diacetylzephyranthine	Up	0
	meta_653	4-Hydroxyphenylacetonitrile triacetylrhamnoside	Up	0
	meta_671	Tryprostatin A	Up	0
	meta_674	Pro-Ser	Up	0
	meta_690	Abiraterone sulfate	Up	0
	meta_706	*N*-Arachidonyl dopamine	Down	0
	meta_740	Retinyl beta-glucuronide	Up	0
	meta_770	1-*O*-Hexadecyl-lyso-sn-glycero-3-phosphocholine	Down	0
	meta_808	1-*O*-Octadecyl-sn-glyceryl-3-phosphorylcholine	Down	0
	meta_816	Nb-trans-Feruloylserotonin glucoside	Up	0
	meta_873	Ile-Phe	Up	0
	meta_877	Nb-trans-Feruloylserotonin glucoside	Up	0
	meta_962	Quercetin 7-glucuronide 3-rhamnoside	Up	0
	meta_1032	Norbadione A	Down	0
	meta_1113	Torvoside E	Up	0
	meta_1139	Phyllanthusol B	Up	0
	meta_1154	PC[18:2(9Z,12Z)/20:1(11Z)]	Up	–
pos	meta_10	3-Hydroxyisovaleric acid	Down	0
	meta_11	Ethylenethiourea	Down	0
	meta_38	l-Threonine	Up	10
	meta_39	Purine	Up	0
	meta_53	l-Leucine	Up	11
	meta_54	l-Asparagine	Up	7
	meta_57	d-Aspartic acid	Up	1
	meta_77	l-Glutamine	Up	16
	meta_81	Methyl 4-(methylthio)butyrate	Up	0
	meta_82	l-Methionine	Up	8
	meta_93	Orotate	Up	2
	meta_96	l-dihydroorotate	Up	2
	meta_101	2-Oxoadipic acid	Up	6
	meta_111	Phenylpyruvate	Up	5
	meta_126	Cytosine	Up	2
	meta_139	*N*-carbamoyl-l-aspartate	Up	3
	meta_145	Aspirin	Up	1
	meta_148	l-Tyrosine	Up	19
	meta_177	1-Isothiocyanato-4-phenylbutane	Up	0
	meta_185	Dihydrolipoate	Down	–
	meta_208	l-Tryptophan	Up	12
	meta_219	d-Ribulose 5-phosphate	Down	6
	meta_220	Benzyl Benzoate	Up	0
	meta_234	2-Iodophenol	Up	0
	meta_238	Pseudoecgonine	Up	0
	meta_278	1-(2,4,5-Trimethoxyphenyl)-1,2-propanedione	Up	0
	meta_287	Methasulfocarb	Up	0
	meta_299	d-Biotin	Down	4
	meta_308	(E)-2-(2-Furyl)-3-(5-nitro-2-furyl)acrylamide	Up	0
	meta_332	3-methylcytidine	Down	0
	meta_339	Primaquine	Up	0
	meta_344	Carbadox	Up	0
	meta_377	d-Glucuronic acid 1-phosphate	Up	0
	meta_394	2-Iodophenol	Up	0
	meta_408	Xanthosine	Down	3
	meta_460	Malathion monocarboxylic acid	Up	0
	meta_464	Eicosapentaenoic Acid	Up	1
	meta_467	Hesperetin	Up	0
	meta_498	Hippeastrine	Down	0
	meta_509	Adipostatin A	Up	0
	meta_523	*N*2-(3-Hydroxysuccinoyl)arginine	Down	0
	meta_528	(4Z,7Z,10Z,13Z,16Z,19Z)-4,7,10,13,1 6,19-Docosahexaenoic acid	Up	0
	meta_533	(7Z,10Z,13Z,16Z,19Z)-Docosapentaenoic acid	Up	0
	meta_536	Adrenic acid	Up	1
	meta_537	l-Thyronine	Up	0
	meta_538	2-Methyl-1,4-naphthalenediol bis(dihydrogen phosphate)	Up	0
	meta_543	Cappariloside A	Up	0
	meta_545	Docosatrienoic Acid	Up	0
	meta_551	Versicolorin A	Up	0
	meta_610	Pyriminobac-methyl	Up	0
	meta_617	9-Aminocamptothecin	Up	0
	meta_657	Pioglitazone	Down	0
	meta_660	Salbutamol 4-*O*-sulfate	Up	0
	meta_672	Methylsyringin	Up	0
	meta_690	Shanzhiside	Up	0
	meta_731	Azidocillin	Up	0
	meta_750	Benzbromarone	Up	0
	meta_763	3-*O*-Caffeoyl-1-*O*-methylquinic acid	Up	0
	meta_778	Phosacetim	Up	0
	meta_785	Margrapine A	Up	0
	meta_806	CDP-ethanolamine	Up	2
	meta_807	Mollicellin E	Up	0
	meta_982	Apramycin	Up	0
	meta_1107	Dimoracin	Up	0
	meta_1113	Lanceotoxin A	Up	0
	meta_1115	Isotetrandrine	Up	0
	meta_1120	Geranyl diphosphate	Up	3
	meta_1154	*N*-Lignoceroylsphingosine	Up	0
	meta_1168	Streptomycin 6-phosphate	Up	0
	meta_1215	Adouetine Z	Up	0
	meta_1230	PE(P-18:0/14:0)	Down	0
	meta_1239	PE[P-18:1(9Z)/16:1(9Z)]	Up	0
	meta_1265	PE[P-18:1(11Z)/20:5(5Z,8Z,11Z,14Z,17Z)]	Up	0
	meta_1296	PE[22:6(4Z,7Z,10Z,13Z,16Z,19Z)/P-18:1(11Z)]	Up	0
	meta_1327	Quinquenoside F1	Up	0
	meta_1332	Punigluconin	Up	0
	meta_1376	Elatoside E	Up	0
	meta_1377	PI[20:3(8Z,11Z,14Z)/18:2(9Z,12Z)]	Up	0
	meta_1388	Bacteriochlorophyll b	Up	0

Selection is based on an MS2 score greater than 0.75

### Cluster analysis of differential metabolites

**Fig. 5:**
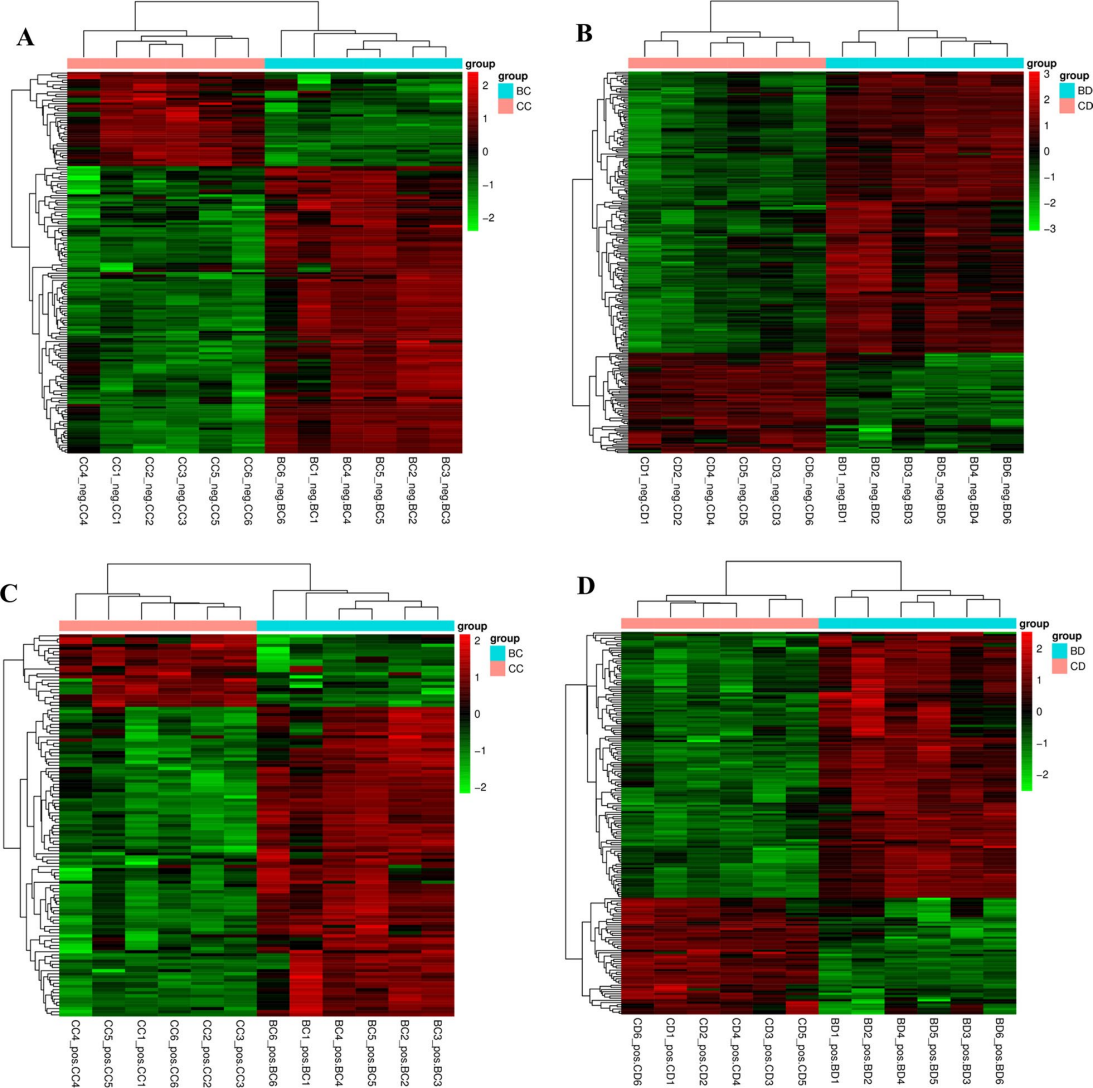
Cluster analysis of differential metabolites in *T. annulata*-transformed cells treated with DMSO and buparvaquone. **a**, **b** 48 h and 72 h (neg); **c**, **d** 48 h and 72 h (pos). log_2_ conversion and normalization were carried out for cluster analysis

Cluster analysis of the detected differential metabolites can further determine the metabolites related to biological diseases and the change trend of differential metabolites in each group of samples. The results are shown in Fig. 5. It can be seen that the differential metabolites had great significance for identifying the experimental and control groups. Judging from the binary tree discrimination, the differences between groups were significantly larger than those within groups, and the parallelism between groups was good, indicating that the drugs would induce significant metabolic differences in the treatment of parasite-infected host cells. Moreover, in each mode, the cluster analysis results of metabolites in the heat map show significant response differences, which are displayed as corresponding colors. These all illustrate that drug treatment can induce significant metabolic differences in host cells. To further analyze specific metabolic function and pathway differences, we performed subsequent KEGG analysis based on differential metabolites.

### KEGG pathway analysis

**Fig. 6:**
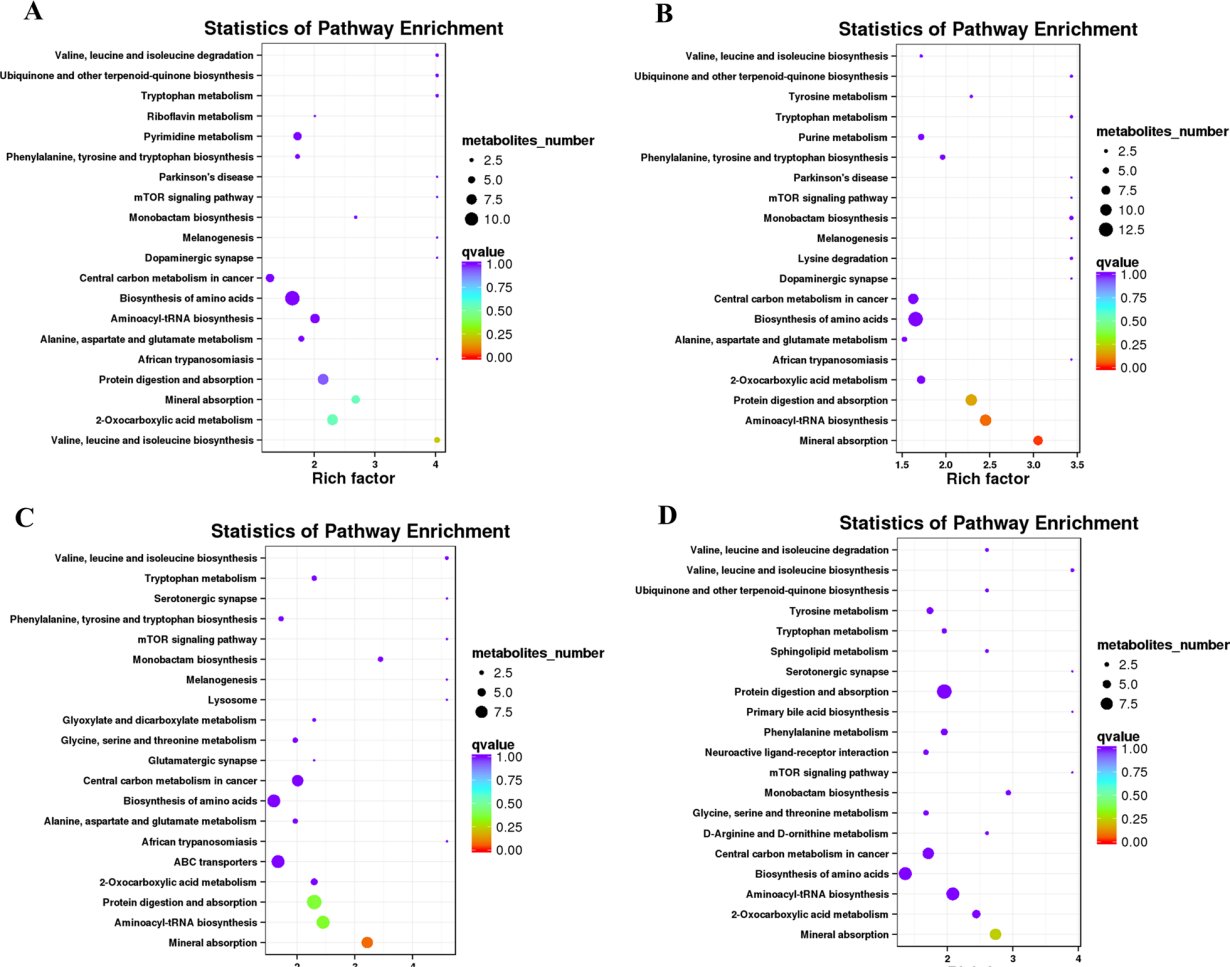
KEGG enrichment analysis of metabolites in host cells after buparvaquone treatment (DMSO is the control treatment group). **a**, **b** Metabolic differences at 48 h and 72 h in negative ion mode. **c**, **d** Metabolic differences at 48 h and 72 h in positive ion mode. The size of the midpoint in the figure represents the number of significantly different metabolites enriched in the corresponding pathway. The color of the point is related to the *Q*-value. The abscissa represents the enrichment factor and the ordinate represents the metabolic pathway

All KEGG-enriched pathways identified were analyzed (Additional file 1: Figure S3), and the results showed that the main pathway belonged to the metabolic part. On this basis, a bubble chart was made for the metabolic part. The identified differential metabolites were annotated with KEGG and then classified according to the annotation results. Under the two models, the differential metabolite classification mainly focused on the metabolic pathways, protein digestion and absorption, and the biosynthesis of amino acids (Additional file 1: Figure S3). With the prolonging of drug treatment time, the differential metabolites in the biosynthesis of amino acids, metabolic pathway, protein digestion and absorption, and central carbon metabolism in cancer increased, and the proportion of these differential metabolites was also relatively high. In neg ion mode, when the treatment time was 48 h (Fig. 6a), four metabolic pathways showed lower *P*-values and higher pathway impact. At 72 h (Fig. 6b), there were three metabolic pathways with lower *P*-values and higher pathway effects. In these two different time periods, the pathway effect of mineral absorption and protein digestion and absorption was greater. Furthermore, biosynthesis involving amino acids occupied the largest number of metabolites. At 48 h in pos ion mode (Fig. 6c), there were three metabolic pathways with lower *P*-value and higher pathway effect. At 72 h (Fig. 6d), there was only one metabolic pathway, mineral absorption, which was consistent with the above scenario. In this model, amino acid biosynthesis, proteome digestion and absorption, and aminoacyl-transfer RNA (tRNA) biosynthesis occupy more metabolites. However, whether in neg ion mode or pos ion mode, the pathway had a greater impact. From Table 3 and Table S2 (Additional file 2), its enrichment factors (neg) were 2.68 and 3.05, with six and eight differential metabolites, respectively. The enrichment factors (pos) were 3.22 and 2.74, respectively. The number of differential metabolites remained unchanged, showing that this metabolic pathway played a great role in the normal physiological function of cells. Although there are certain differences in the detection of metabolites between the two test modes, the main differences are still concentrated in the two pathways of amino acid biosynthesis, protein digestion and absorption. This may be the key to subsequent analysis of how BW720c treats parasite-infected host cells and inhibits *T. annulata* proliferation.

**Table 3:** Enrichment factor, *Q*-value and differential metabolite for each metabolic pathway at 48 h and 72 h for both pos and neg modes

Time	Number	Metabolic pathway	Enrichment factor	*Q*-value	Differential metabolite
48 h (neg)	1	Valine, leucine, and isoleucine biosynthesis	4.03	0.22	4
	2	2-Oxocarboxylic acid metabolism	2.30	0.55	8
	3	Mineral absorption	2.68	0.55	6
	4	Protein digestion and absorption	2.15	0.93	8
72 h (neg)	1	Mineral absorption	3.05	0.02	8
	2	Aminoacyl-tRNA biosynthesis	2.45	0.07	10
	3	Protein digestion and absorption	2.29	0.16	10
48 h (pos)	1	Mineral absorption	3.22	0.07	7
	2	Aminoacyl-tRNA biosynthesis	2.45	0.35	8
	3	Protein digestion and absorption	2.30	0.36	9
72 h (pos)	1	Mineral absorption	2.74	0.24	7

## Discussion

Tropical theileriosis has caused great economic losses in the livestock industry, with approximately 250 million cattle being threatened by the disease globally [31]. A previous study showed that the unlimited tumor-like proliferation of lymphocytes could be induced by the culture of schizont in vitro [15], and Lira et al. reported that metabolic changes were characteristic of tumor cells [10, 32], which provided the ideas for this study. When the antitheilerial drug buparvaquone (BW720c) acts on the transformed cells, it can terminate the longevity state of the cells and allow them to re-enter the normal apoptotic program. Metabolomics is an important method for studying the interaction between the pathogen and the host. Studying the effect of *T. annulata* on cell metabolomics during cell transformation under the action of BW720c will provide new insights and enable a deeper understanding of the mechanism of *T. annulata* in bovine cell transformation. In this study, the LC–QTOF-MS technique was used to examine the metabolomics of host cells following drug-mediated parasite infection. With this approach, it was possible not only to obtain the metabolic profiles of host cells in different periods after drug treatment, but also to identify the metabolites and their associated metabolic pathways. PCA and OPLS-DA showed that the metabolites of cells after drug treatment had very significant time-dependent changes. With the prolonged treatment time of BW720c, the aggregation within and the separation between groups increased. This may be related to the process of destabilization and restoration of cell metabolism after the action of the drug [33]. Subsequent analysis showed that the number of upregulated differential metabolites was greater than that of downregulated differential metabolites, and the number of differential metabolites increased over time. This indicates that drug-induced variant responses in *T. annulata*-infected cell metabolism are time-dependent. Also, the differences in metabolites between the control and experimental groups increased with time. In addition, the KEGG enrichment analysis results indicated that the identification of differential metabolites found that most of them were amino acids or their derivatives.

The metabolism of amino acids in vivo is very complex, and its biosynthesis is one of the basic metabolic pathways. The balance of amino acids is very important to maintain the normal growth and development of the body. In the healthy state, the level of amino acids is in dynamic equilibrium, however, when *T. annulata* infects cells, the metabolism of amino acids is disordered. In tumor cells, the de novo serine synthesis pathway is an important branch of glycolysis. Over-activation of the serine/glycine metabolic pathway is considered to be involved in carcinogenesis [34, 35]. In this study, l-serine was upregulated, which can be considered as over-activation. Serine metabolism provides essential precursors such as amino acids and nucleotides, controls the antioxidant and methylation capacity of cells, and promotes the growth of cancer cells [36]. l-Leucine was an upregulated differential metabolite in two modes. Leucine was involved in 11 metabolic pathways, such as central carbon metabolism in cancer, aminoacyl tRNA biosynthesis, mammalian target of rapamycin (mTOR) signaling pathway, biosynthesis of amino acids, protein digestion, and absorption between the positive and negative ion modes. Leucine can regulate autophagy activity in mTORC1-dependent or -independent ways [37, 38] and significant earlier studies further confirmed that mTORC1 can promote the translation efficiency of mitochondrial fission process 1 mRNA, and thus regulate the mitosis and apoptosis of mitochondria [39–41]. Due to the high concentration of leucine in cells, the mTORC1 pathway can be activated [42, 43]. Therefore, it is speculated that the programmed cell apoptosis accompanying BW720c treatment may be related to the elevated leucine level. Glutamine and glutamic acid have similar biological functions. They can participate in the biosynthesis of α-ketoglutarate and can supplement citric acid, malic acid, and other tricarboxylic acid cycle intermediates [44]. Leucine can be a source of glutamine synthesis. 3-Hydroxyisovalerate, a common cellular metabolite, is a by-product of the leucine degradation pathway, and its production begins with the conversion of 3-methylcrotonyl-CoA in the mitochondria. The downregulation of 3-hydroxyisovalerate implies metabolic dysregulation caused by excessive accumulation of leucine. In this study, the trend of their changes was the same, and both of them became upregulated metabolites after drug treatment. At 72 h of drug treatment, the levels of leucine and glutamine in the experimental group were 3.92 times and 3 times as high as those in the control group, respectively, in neg ion mode, while in pos ion mode, leucine and glutamine were 2.86 and 3.16 times as high as those in the control group. The content of these two amino acids changed significantly after treatment. Previous studies have shown that the in vitro proliferation of human liver cancer cells leads to a significant increase in the consumption of glutamine, threonine, and arginine [45, 46]. Therefore, it is speculated that the decrease in cell requirements for glutamine and threonine after drug treatment may lead to an increase in these two amino acids.

l-Tyrosine was upregulated. According to the study by Si et al., l-tyrosine is related to the number of thymic mast cells and can promote the formation of thymic mast cell maturation and development. l-Tyrosine can be directly used as a substrate for melanin synthesis; in contrast, mast cells may have sufficient substrates to synthesize and secrete catecholamine or serotonin [47]. Meanwhile, inhibition of 5-HT production in peripheral blood can effectively reduce lung group neuroendocrine tumor (NET) generation, thereby reducing lung injury [48]. If infected cells were treated with serotonin inhibitors, it could have the potential to promote apoptosis. Tyrosine kinases, which play an essential role in growth factor signaling regulation, are significant targets for antitumor and antileukemia agents. It is suspected that tyrosine inhibitors can also be used to promote apoptosis of infected cells. Phospholipids are an important cell component, including phosphatidylcholine (PC), phosphatidylinositol (PI), phosphatidic acid (PA), and phosphatidylethanolamine (PE). Our results were consistent with that in neg ion mode (72 h) in which PC, PI, and PE all increased. It was shown that treatment with the drug caused damage to the host cell membrane/parasite system. d-Ribulose 5-phosphate was downregulated in the biosynthesis of amino acids at 72 h of drug treatment. In addition, it was shown to participate in the pentose phosphate pathway (PPP), carbon metabolism, and vitamin B6 metabolism. In recent years, it has been reported that tumor cells rely on glycolysis to obtain energy [49], but according to the research by Wood [50], tumor cells need to synthesize a large number of nucleotides and lipids for rapid division and proliferation, and approximately 85% of pentose for DNA synthesis was provided by PPP. A clinical study in 2014 also confirmed the existence of high flow PPP in human cancer cells [51]. All these studies indicate that PPP is the energy source of tumor cells. In this study, with the prolongation of drug treatment time, the parasite was cleared at 72 h, so no additional energy was needed to support the survival of the parasite. d-Ribulose 5-phosphate is the final product of PPP, and its downregulation also showed the weakening of the pathway. Due to the detection results, it is also speculated that PPP is one of the energy sources of tumor-like cells caused by *T. annulata*. PPP pathway not only provides the energy needed for cell survival, but also produces NADPH, which can be used as a protection to eliminate the harm of free radicals produced by *T. annulata*. Based on this, the key enzyme of PPP in the cell invaded by *T. annulate* can be blocked, just as the key enzyme of glycolysis can be blocked to inhibit this pathway and thereby inhibit tumors [52]. Boros et al. have shown that targeting PPP with dehydroepiandrosterone (DHEA) and thiamine oxide can inhibit G6PD and TKT, respectively, which have antitumor effects [53]. Therefore, this may be a direction for treatment of the disease.

In the pos mode (72 h), l-carnitine (LC) and dihydrolipoate were downregulated. Hypoxanthine was upregulated. LC is an amino acid-like substance that is required for β-oxidation of long-chain fatty acids and transport of fatty acids to the inner mitochondrial membrane by carnitine palmitoyltransferase I (CPT1) [54]. Therefore, l-carnitine plays a crucial role in energy metabolism. l-carnitine deficiency may lead to severe dysfunction of intracellular mitochondria and the failure of cellular lipid utilization [55]. l-carnitine deficiency leads to impaired production of beta-oxidation, which in turn induces mitochondrial dysfunction and leads to altered metabolism and multiple organ dysfunction. In addition, cancer patients consume l-carnitine during chemotherapy [56] and it has also been found that LC can regulate the process of apoptosis and DNA damage [57, 58]. In this study, LC was downregulated at 72 h of drug treatment. It is speculated that BW70c treatment can inhibit host cell LC production and severely impair β-oxidation, leading to abnormalities in energy metabolism that further induce direct host cell death [59]. Dihydrolipoic acid (DHLA), an active form of lipoic acid (LA), is a powerful electron donor induced by lipoic acid amide dehydrogenase in cells [60]. It has been found to stabilize lysosomal membranes, reduce oxidative stress, and exert beneficial effects in rat models of various diseases [61]. The downregulation of DHLA content in host cells after BW720c treatment may presuppose lysosomal damage in host cells or parasites and be a potential risk of high oxidative stress, which will increase host or parasite apoptosis risk. This is consistent with the increased detection of the metabolite phospholipids described above. Hypoxanthine is a metabolite of nucleoside and an important alkaloid purine [62]. In this study, under the pos ion mode (72 h), the amount of the substance was upregulated and involved in metabolic pathways and purine metabolism, which showed that the substance was of great significance to the normal physiology of cells. It has been reported that *Toxoplasma gondii* cannot synthesize purine, and it is very dependent on the enzymes and intermediates in the purine salvage pathway [63]. The purines of infected cells are upregulated after being treated with drugs, so we speculate that *T. annulata* may not have the ability to synthesize purines, and thus they will be upregulated after parasite removal. In addition, upregulation of purines may also be due to changes in purine content in host cells due to the degradation of DNA and RNA in parasite cells after death from the drug [64]. Pro-Glu, Pro-Thr, and Thr-Asp were detected in cells in pos mode. These dipeptides are upregulated in the cell and not annotated on the KEGG pathway, so we speculated that this may be the product of degradation of the parasite protein after drug clearance. In neg ion mode, no upregulated dipeptides were observed, which may have been caused by the different detection modes. In summary, through the analysis of different metabolites under the treatment of positive and negative ions for 72 h, it can be concluded that the effects of drugs on parasite-infected cells mainly focus on interfering with amino acid metabolism and energy metabolism to achieve a sufficient effect.

## Conclusions

In this work, the metabolomics of *T. annulate*-infected cells were tested under BW720c treatment. The results showed that, compared with DMSO treatment, BW720c could significantly alter the host cell metabolome, resulting in significant differences; and the differences also expanded with the prolongation of drug treatment time. In addition, the analysis of differential metabolites revealed that downregulation occurred for 3-hydroxyisovalerate (a downstream metabolite of leucine metabolism), l-carnitine (LC) and DHLA, and other metabolites; upregulation occurred for the metabolism of leucine, inosine, and hypoxanthine. Through the analysis of differential metabolites, the poor retention of nitrogen, the initiation of apoptosis, and cell carcinogenesis caused by the abnormality of the leucine metabolic pathway are the potential mechanisms of action. This article provides ideas for further elucidating the mechanism of the transformation of cells of *T. annulata* under BW720c treatment. However, the lack of comparison between infected and uninfected cells remains a shortcoming of the study.

## Supplementary Information

**Additional file 1: Table S1.** Mobile phase conditions of liquid chromatography. **Figure S1.** Total ion chromatogram (TIC) of the quality control sample. **Figure S2.** PCA of all samples. **Figure S3.** KEGG enrichment analysis.

**Additional file 2: Table S2.** All differential metabolites at 48 h and 72 h in both modes (neg and pos).

## Data Availability

All data generated during this study are included in this published article and its Additional information files (Additional Figures S1–S3 and Tables S1, S2).

## Abbreviations

BW720c: Buparvaquone
DMSO: Dimethyl sulfoxide
QC: Quality control
PCA: Principal component analysis
OPLS-DA: Orthogonal partial least squares analysis
FC: Fold change
mTOR: Mammalian target of rapamycin
TIC: Total ion chromatogram
LC–QTOF-MS: Liquid chromatography-quadrupole time-of-flight mass spectrometry
