# Supplementary material for: Metabolomic profiling of bovine leucocytes transformed by Theileria annulata under BW720c treatment
Source: Parasit Vectors. 2022 Oct 5;15:356. doi: 10.1186/s13071-022-05450-0 (PMC9533618; doi:10.1186/s13071-022-05450-0)
Supplement: Supplementary file 1 — Additional file 1: Table S1. Mobile phase conditions of liquid chromatography. Figure S1. Total ion chromatogram (TIC) of the quality control sample. Figure S2. PCA of all samples. Figure S3. KEGG enrichment analysis. [file 13071_2022_5450_MOESM1_ESM.docx]

**Supporting Information**

Metabonomic profiling of bovine leucocytes transformed by *Theileria annulata* under BW720c treatment

**Section 1. LC-QTOF-MS analysis conditions**

**1. 1.** **Liquid chromatography conditions**

In this experiment, water (25mM ammonium acetate and 25mM ammonia) and acetonitrile were used as mobile phase. The setting of mobile phase is shown in Table 4. The chromatographic column used is UPLC BEH amide (1.7 μm * 2.1 * 100mm) purchased from waters.

**Table. S1** Mobile phase conditions of liquid chromatography.

| Time  （min） | Current Speed  （μL/min） | A% water（25mM ammonium  acetate and 25mM ammonia） | B% acetonitrile |
| --- | --- | --- | --- |
| 0 | 500 | 5 | 95 |
| 0.5 | 500 | 5 | 95 |
| 7 | 500 | 35 | 65 |
| 8 | 500 | 60 | 40 |
| 9 | 500 | 60 | 40 |
| 9.1 | 500 | 5 | 95 |

**1. 2. Mass spectrum conditions**

AB 5600 triple TOF mass spectrometer can collect primary and secondary mass spectrum data based on IDA function under the control of control software (Analyst TF 1.7, AB Sciex). In each data collection cycle, molecular and ions with the strongest strength, which is greater than 100 were selected to collect the corresponding secondary mass spectrometry data. ESI ion source parameters are set as follows: atomization pressure (GS1): 60Psi, auxiliary pressure: 60Psi, air curtain pressure: 35Psi, temperature: 650 C, spray voltage: 5000V (pos mode) or -4000V (neg mode).

**1. 3. Methodological investigation**

LC-MS is a very complex and precise system, which will be affected by temperature, humidity, vibration, circuit board aging and other objective factors, resulting in a certain degree of response signal fluctuation. In the test process, the stability of the instrument is very important to the test results. Real time monitoring the stability of the instrument and whether the signal is normal is helpful to find out the abnormality in time and eliminate the problem as soon as possible, so as to ensure the quality of the final collected data. Figure.1 is quality control results.

**1. 4. Metabolite quantification**

The protowizard software was used to transform the original mass spectrum into mzXML format. Then XCMS is used to do the work of retention time correction, peak recognition, peak extraction, peak integration, peak alignment and so on. Minfrac is set to 0, and cutoff is set to 0.6. At the same time, the R program package and the secondary mass spectrometry database were used to identify the peaks. Then we preprocess the data as the following way: filtering the single peak, and only keeping the peak area data with single group empty value no more than 50% or all groups empty value no more than 50%; the missing values in the original data are filled by simulation, and the numerical simulation method is the minimum value one- half method; the total ion current of each sample is used for normalization.

**Section 2. LC-QTOF-MS analysis results**

**Quality control sample results.**

We analyzed the total ion chromatogram of quality control samples. As can be seen from Fig.S1 that the peak retention time and peak area of TIC of six QC samples overlapped well, and there was no peak displacement, indicating that the instrument had good stability.


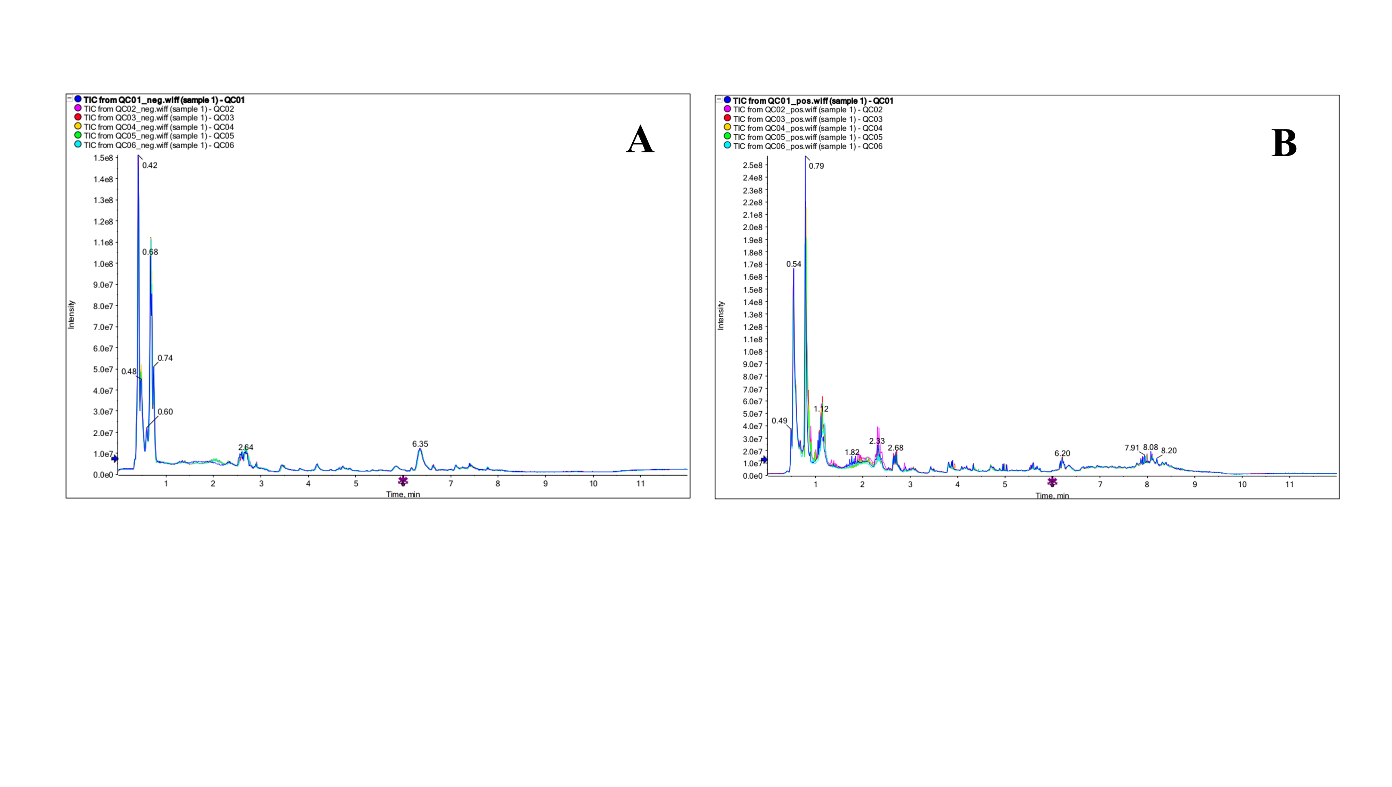


**Figure. S1** total ion chromatogram (TIC) of quality control sample. A: negative ion mode (ESI-) and B: positive ion mode (ESI+). X axis represents peak time, Y axis represents intensity.


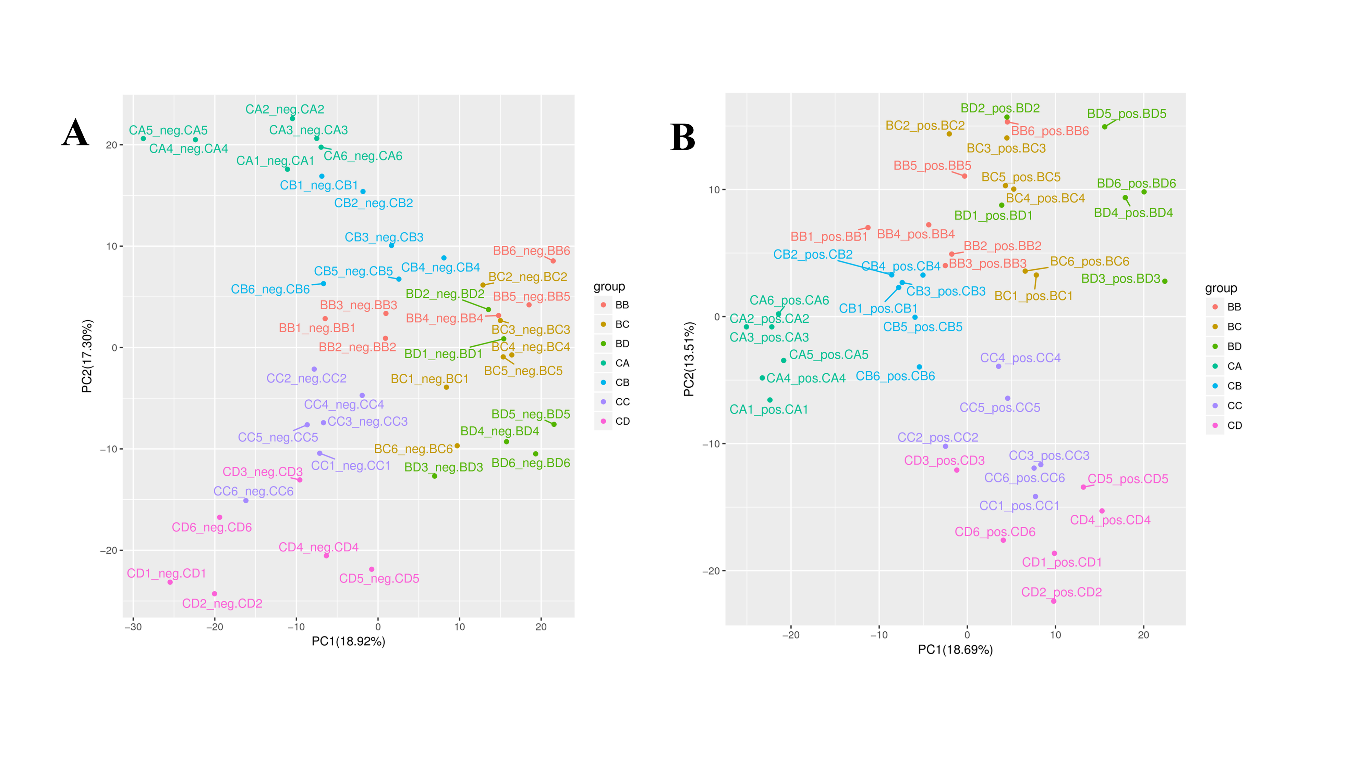


**Figure. S2** PCA analysis of all samples. A neg mode; B pos mode. BB, BC, BD represent BW720c treatments 24, 48 and 72 h, respectively; CA, CB, CC, and CD represent DMSO treatments 0, 24, 48 and 72 h, respectively.

**
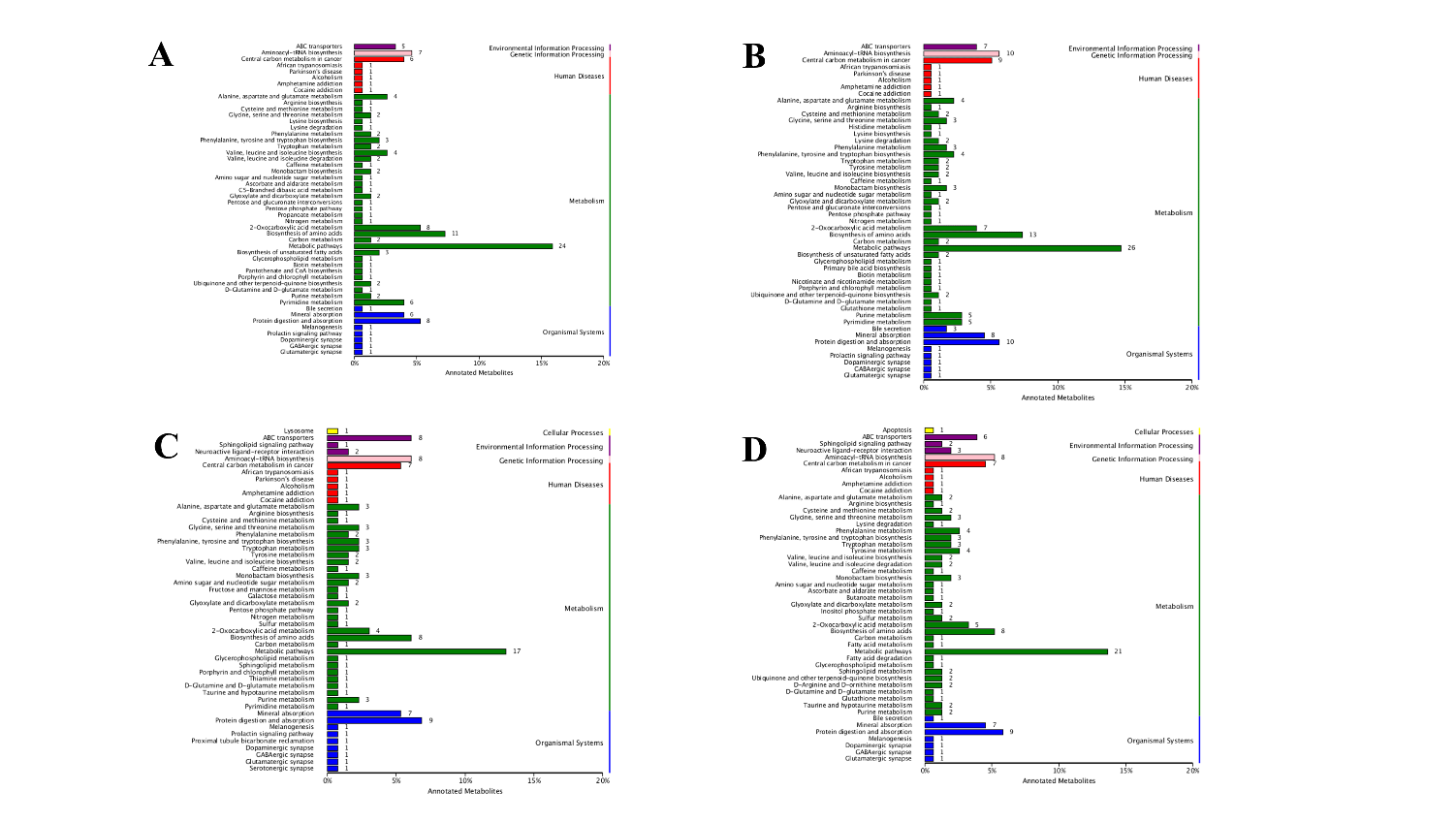
**

**Figure. S3** KEGG enrichment analysis. A-B: 48 h, 72 h (neg); C-D: 48 h, 72 h (pos).
