# Supplementary material for: Metabolomic profiling of bovine leucocytes transformed by Theileria annulata under BW720c treatment
Source: Parasit Vectors. 2022 Oct 5;15:356. doi: 10.1186/s13071-022-05450-0 (PMC9533618; doi:10.1186/s13071-022-05450-0)
Supplement: Supplementary file 2 — Additional file 2: Table S2. All differential metabolites at 48 h and 72 h in both modes (neg and pos). [file 13071_2022_5450_MOESM2_ESM.docx]

**Supporting Information Table S2**

**Table S2** All differential metabolites at 48 hours and 72 hours in both modes(neg and pos)

| Mode | Time | ID | Metabolite | Fold_change | VIP | Regulated | KEGG annotation |
| --- | --- | --- | --- | --- | --- | --- | --- |
| pos | 48h | meta_6 | Unmapped | 3.47 | 1.73 | up | 0 |
|  |  | meta_10 | 3-Hydroxyisovaleric acid | 0.40 | 1.78 | down | 0 |
|  |  | meta_11 | Ethylenethiourea | 0.39 | 1.38 | down | 0 |
|  |  | meta_14 | 2-hydroxy-butanoic acid | 0.47 | 1.54 | down | 1 |
|  |  | meta_17 | p-Cresol | 2.28 | 1.68 | up | 2 |
|  |  | meta_24 | Uracil | 0.49 | 1.73 | down | 4 |
|  |  | meta_35 | Diazenedicarboxamide | 2.96 | 1.43 | up | 0 |
|  |  | meta_38 | L-Threonine | 2.07 | 1.88 | up | 10 |
|  |  | meta_39 | Purine | 2.69 | 1.69 | up | 0 |
|  |  | meta_47 | Unmapped | 0.49 | 1.21 | down | 0 |
|  |  | meta_48 | Mesaconic acid | 0.26 | 1.82 | down | 6 |
|  |  | meta_50 | ketoisocaproic acid | 0.48 | 1.23 | down | 5 |
|  |  | meta_53 | L-Leucine | 2.43 | 1.90 | up | 11 |
|  |  | meta_54 | L-Asparagine | 2.48 | 1.45 | up | 7 |
|  |  | meta_57 | D-Aspartic acid | 4.47 | 1.78 | up | 1 |
|  |  | meta_77 | L-Glutamine | 2.73 | 1.89 | up | 16 |
|  |  | meta_80 | (S)-2-Hydroxyglutarate | 0.22 | 1.79 | down | 0 |
|  |  | meta_81 | Methyl 4-(methylthio)butyrate | 2.13 | 1.89 | up | 0 |
|  |  | meta_82 | L-Methionine | 2.08 | 1.93 | up | 8 |
|  |  | meta_93 | Orotate | 4.90 | 1.92 | up | 2 |
|  |  | meta_96 | L-dihydroorotate | 17.87 | 1.88 | up | 2 |
|  |  | meta_101 | 2-Oxoadipic acid | 2.92 | 1.84 | up | 6 |
|  |  | meta_111 | Phenylpyruvate | 3.21 | 1.67 | up | 5 |
|  |  | meta_126 | Cytosine | 2.15 | 1.52 | up | 2 |
|  |  | meta_139 | N-carbamoyl-L-aspartate | 9.57 | 1.90 | up | 3 |
|  |  | meta_145 | Aspirin | 3.63 | 1.62 | up | 1 |
|  |  | meta_148 | L-Tyrosine | 2.17 | 1.87 | up | 19 |
|  |  | meta_176 | Unmapped | 2.57 | 1.51 | up | 0 |
|  |  | meta_177 | 1-Isothiocyanato-4-phenylbutane | 2.51 | 1.85 | up | 0 |
|  |  | meta_208 | L-Tryptophan | 2.45 | 1.89 | up | 12 |
|  |  | meta_218 | Elaeokanine C | 0.39 | 1.27 | down | 0 |
|  |  | meta_219 | D-Ribulose 5-phosphate | 0.46 | 1.70 | down | 6 |
|  |  | meta_220 | Benzyl Benzoate | 2.05 | 1.64 | up | 0 |
|  |  | meta_234 | 2-Iodophenol | 5.42 | 1.85 | up | 0 |
|  |  | meta_237 | 5-(2-Hydroxyethyl)-4-methylthiazole acetate | 2.54 | 1.33 | up | 0 |
|  |  | meta_238 | Pseudoecgonine | 2.08 | 1.47 | up | 0 |
|  |  | meta_239 | N-Acetyl-D-glucosamine | 2.08 | 1.40 | up | 3 |
|  |  | meta_273 | Unmapped | 3.29 | 1.89 | up | 0 |
|  |  | meta_278 | 1-(2,4,5-Trimethoxyphenyl)-1,2-propanedione | 2.71 | 1.66 | up | 0 |
|  |  | meta_287 | Methasulfocarb | 3.30 | 1.68 | up | 0 |
|  |  | meta_299 | D-Biotin | 0.40 | 1.62 | down | 4 |
|  |  | meta_308 | (E)-2-(2-Furyl)-3-(5-nitro-2-furyl)acrylamide | 2.77 | 1.90 | up | 0 |
|  |  | meta_328 | Unmapped | 2.25 | 1.66 | up | 0 |
|  |  | meta_329 | 2'-Deoxysepiapterin | 2.34 | 1.43 | up | 0 |
|  |  | meta_332 | 3-methylcytidine | 0.06 | 1.81 | down | 0 |
|  |  | meta_339 | Primaquine | 2.06 | 1.75 | up | 0 |
|  |  | meta_344 | Carbadox | 2.34 | 1.65 | up | 0 |
|  |  | meta_377 | D-Glucuronic acid 1-phosphate | 2.55 | 1.62 | up | 0 |
|  |  | meta_384 | Unmapped | 2.22 | 1.26 | up | 0 |
|  |  | meta_394 | 2-Iodophenol | 6.95 | 1.90 | up | 0 |
|  |  | meta_408 | Xanthosine | 0.41 | 1.35 | down | 3 |
|  |  | meta_410 | Phenytoin catechol | 2.35 | 1.60 | up | 0 |
|  |  | meta_417 | Tricetanidin | 2.12 | 1.53 | up | 0 |
|  |  | meta_422 | Orotidine | 0.37 | 1.78 | down | 0 |
|  |  | meta_460 | Malathion monocarboxylic acid | 5.89 | 1.90 | up | 0 |
|  |  | meta_464 | Eicosapentaenoic Acid | 3.97 | 1.81 | up | 1 |
|  |  | meta_467 | Hesperetin | 3.21 | 1.89 | up | 0 |
|  |  | meta_498 | Hippeastrine | 0.11 | 1.77 | down | 0 |
|  |  | meta_509 | Adipostatin A | 2.77 | 1.96 | up | 0 |
|  |  | meta_522 | Butote | 3.37 | 1.87 | up | 0 |
|  |  | meta_523 | N2-(3-Hydroxysuccinoyl)arginine | 0.44 | 1.84 | down | 0 |
|  |  | meta_528 | (4Z,7Z,10Z,13Z,16Z,19Z)-4,7,10,13,1 6,19-Docosahexaenoic acid | 4.61 | 1.87 | up | 0 |
|  |  | meta_533 | 7Z, 10Z, 13Z, 16Z, 19Z-Docosapentaenoic acid | 4.44 | 1.87 | up | 0 |
|  |  | meta_536 | Adrenic Acid | 2.26 | 1.86 | up | 1 |
|  |  | meta_537 | L-Thyronine | 3.11 | 1.43 | up | 0 |
|  |  | meta_538 | 2-Methyl-1,4-naphthalenediol bis(dihydrogen phosphate) | 11.96 | 1.63 | up | 0 |
|  |  | meta_543 | Cappariloside A | 2.22 | 1.58 | up | 0 |
|  |  | meta_545 | Docosatrienoic Acid | 3.13 | 1.87 | up | 0 |
|  |  | meta_551 | Versicolorin A | 22.37 | 1.91 | up | 0 |
|  |  | meta_574 | Adenosine monophosphate (AMP) | 0.46 | 1.29 | down | 0 |
|  |  | meta_589 | Unmapped | 2.25 | 1.64 | up | 0 |
|  |  | meta_597 | L-Galactono-1,4-lactone | 2.31 | 1.66 | up | 2 |
|  |  | meta_602 | Bufanolide | 3.25 | 1.80 | up | 0 |
|  |  | meta_610 | Pyriminobac-methyl | 2.34 | 1.74 | up | 0 |
|  |  | meta_614 | Unmapped | 0.44 | 1.45 | down | 0 |
|  |  | meta_617 | 9-Aminocamptothecin | 2.70 | 1.66 | up | 0 |
|  |  | meta_620 | N-Oleoylethanolamine | 0.46 | 1.43 | down | 0 |
|  |  | meta_622 | Unmapped | 3.45 | 1.80 | up | 0 |
|  |  | meta_626 | Nervonic acid | 2.06 | 1.62 | up | 1 |
|  |  | meta_629 | 3-O-Caffeoyl-1-O-methylquinic acid | 2.03 | 1.48 | up | 0 |
|  |  | meta_630 | 5-O-Feruloylquinic acid | 2.75 | 1.56 | up | 0 |
|  |  | meta_633 | Unmapped | 4.74 | 1.42 | up | 0 |
|  |  | meta_657 | Pioglitazone | 0.09 | 1.77 | down | 0 |
|  |  | meta_660 | Salbutamol 4-O-sulfate | 9.76 | 1.90 | up | 0 |
|  |  | meta_667 | Unmapped | 5.34 | 1.92 | up | 0 |
|  |  | meta_672 | Methylsyringin | 3.20 | 1.72 | up | 0 |
|  |  | meta_686 | Unmapped | 2.40 | 1.58 | up | 0 |
|  |  | meta_690 | Shanzhiside | 2.40 | 1.61 | up | 0 |
|  |  | meta_704 | 8-Acetoxypinoresinol | 2.18 | 1.37 | up | 0 |
|  |  | meta_707 | Unmapped | 2.45 | 1.30 | up | 0 |
|  |  | meta_710 | 11'-Carboxy-gamma-tocotrienol | 4.96 | 1.78 | up | 0 |
|  |  | meta_716 | Unmapped | 2.44 | 1.70 | up | 0 |
|  |  | meta_731 | Azidocillin | 2.03 | 1.73 | up | 0 |
|  |  | meta_750 | Benzbromarone | 2.55 | 1.88 | up | 0 |
|  |  | meta_763 | 3-O-Caffeoyl-1-O-methylquinic acid | 2.40 | 1.74 | up | 0 |
|  |  | meta_773 | 7,11,12-Triacetoxycoumestan | 0.38 | 1.82 | down | 0 |
|  |  | meta_778 | Phosacetim | 3.73 | 1.63 | up | 0 |
|  |  | meta_780 | 7-Chloro-3,3',4',5,6,8-hexamethoxyflavone | 3.10 | 1.40 | up | 0 |
|  |  | meta_785 | Margrapine A | 5.63 | 1.91 | up | 0 |
|  |  | meta_793 | Unmapped | 0.37 | 1.65 | down | 0 |
|  |  | meta_806 | CDP-ethanolamine | 2.23 | 1.85 | up | 2 |
|  |  | meta_807 | Mollicellin E | 2.46 | 1.92 | up | 0 |
|  |  | meta_814 | Unmapped | 2.15 | 1.62 | up | 0 |
|  |  | meta_840 | Unmapped | 2.37 | 1.95 | up | 0 |
|  |  | meta_878 | Unmapped | 0.41 | 1.64 | down | 0 |
|  |  | meta_879 | Unmapped | 0.50 | 1.76 | down | 0 |
|  |  | meta_915 | LysoPE(18:2(9Z,12Z)/0:0);LysoPE(0:0/18:2(9Z,12Z)) | 2.50 | 1.60 | up | 0 |
|  |  | meta_918 | Carpaine | 2.24 | 1.59 | up | 0 |
|  |  | meta_956 | Unmapped | 2.59 | 1.72 | up | 0 |
|  |  | meta_957 | Unmapped | 3.54 | 1.88 | up | 0 |
|  |  | meta_958 | Unmapped | 3.15 | 1.77 | up | 0 |
|  |  | meta_960 | Buprenorphine | 0.28 | 1.71 | down | 0 |
|  |  | meta_982 | Apramycin | 2.19 | 1.83 | up | 0 |
|  |  | meta_985 | Unmapped | 0.37 | 1.78 | down | 0 |
|  |  | meta_989 | LysoPC(P-18:0) | 0.50 | 1.31 | down | 0 |
|  |  | meta_1019 | Unmapped | 0.11 | 1.57 | down | 0 |
|  |  | meta_1047 | Negletein 6-[rhamnosyl-(1-&gt;2)-fucoside] | 0.49 | 1.30 | down | 0 |
|  |  | meta_1053 | Uridine 5'-diphosphoglucuronic acid (UDP-D-glucuronate) | 0.49 | 1.47 | down | 0 |
|  |  | meta_1055 | Streptomycin | 2.09 | 1.63 | up | 0 |
|  |  | meta_1105 | Cytidine monophosphate N-acetylneuraminic acid | 0.48 | 1.65 | down | 0 |
|  |  | meta_1107 | Dimoracin | 4.19 | 1.82 | up | 0 |
|  |  | meta_1113 | Lanceotoxin A | 3.14 | 1.60 | up | 0 |
|  |  | meta_1115 | Isotetrandrine | 2.21 | 1.53 | up | 0 |
|  |  | meta_1120 | Geranyl diphosphate | 3.71 | 1.85 | up | 3 |
|  |  | meta_1154 | N-Lignoceroylsphingosine | 3.40 | 1.86 | up | 0 |
|  |  | meta_1168 | Streptomycin 6-phosphate | 6.34 | 1.82 | up | 0 |
|  |  | meta_1198 | Dephosphocoenzyme A (Dephospho-CoA) | 0.41 | 1.42 | down | 0 |
|  |  | meta_1206 | 9,12,15-Octadecatrienoic acid 1-[(phosphonoxy)methyl]-1,2-ethanediyl ester | 0.41 | 1.89 | down | 0 |
|  |  | meta_1215 | Adouetine Z | 2.59 | 1.87 | up | 0 |
|  |  | meta_1230 | PE(P-18:0/14:0) | 0.32 | 1.81 | down | 0 |
|  |  | meta_1237 | Lupeoside | 0.43 | 1.73 | down | 0 |
|  |  | meta_1238 | Lupeoside | 0.48 | 1.54 | down | 0 |
|  |  | meta_1239 | PE(P-18:1(9Z)/16:1(9Z)) | 2.09 | 1.30 | up | 0 |
|  |  | meta_1245 | Unmapped | 0.39 | 1.80 | down | 0 |
|  |  | meta_1265 | PE(P-18:1(11Z)/20:5(5Z,8Z,11Z,14Z,17Z)) | 2.50 | 1.47 | up | 0 |
|  |  | meta_1268 | 1-Palmitoyl-2-oleoyl-phosphatidylglycerol | 2.15 | 1.40 | up | 0 |
|  |  | meta_1275 | Unmapped | 0.38 | 1.61 | down | 0 |
|  |  | meta_1296 | PE(22:6(4Z,7Z,10Z,13Z,16Z,19Z)/P-18:1(11Z)) | 3.00 | 1.61 | up | 0 |
|  |  | meta_1306 | Adenosine thiamine triphosphate | 2.47 | 1.86 | up | 0 |
|  |  | meta_1327 | Quinquenoside F1 | 3.04 | 1.81 | up | 0 |
|  |  | meta_1330 | PG(18:1(9Z)/22:5(7Z,10Z,13Z,16Z,19Z)) | 3.35 | 1.81 | up | 0 |
|  |  | meta_1332 | Punigluconin | 2.60 | 1.83 | up | 0 |
|  |  | meta_1346 | PE(22:5(7Z,10Z,13Z,16Z,19Z)/22:5(4Z,7Z,10Z,13Z,16Z)) | 2.02 | 1.77 | up | 0 |
|  |  | meta_1349 | Vinaginsenoside R1 | 4.66 | 1.78 | up | 0 |
|  |  | meta_1352 | PG(18:1(9Z)/22:5(7Z,10Z,13Z,16Z,19Z)) | 5.77 | 1.75 | up | 0 |
|  |  | meta_1356 | Unmapped | 2.91 | 1.74 | up | 0 |
|  |  | meta_1359 | 3-Hydroxybutyryl-CoA | 2.03 | 1.85 | up | 0 |
|  |  | meta_1365 | Unmapped | 4.00 | 1.76 | up | 0 |
|  |  | meta_1366 | Unmapped | 3.18 | 1.60 | up | 0 |
|  |  | meta_1367 | Brevetoxin A | 24.95 | 1.58 | up | 0 |
|  |  | meta_1368 | Koryoginsenoside R1 | 25.30 | 1.60 | up | 0 |
|  |  | meta_1375 | Unmapped | 2.63 | 1.80 | up | 0 |
|  |  | meta_1376 | Elatoside E | 2.61 | 1.76 | up | 0 |
|  |  | meta_1377 | PI(20:3(8Z,11Z,14Z)/18:2(9Z,12Z)) | 2.83 | 1.81 | up | 0 |
|  |  | meta_1381 | Unmapped | 3.11 | 1.82 | up | 0 |
|  |  | meta_1384 | Unmapped | 2.34 | 1.79 | up | 0 |
|  |  | meta_1388 | Bacteriochlorophyll b | 2.90 | 1.79 | up | 0 |
|  |  | meta_1390 | PI(20:4(8Z,11Z,14Z,17Z)/20:1(11Z)) | 2.55 | 1.56 | up | 0 |
|  |  | meta_1393 | Unmapped | 0.41 | 1.36 | down | 0 |
|  |  | meta_1395 | Unmapped | 2.28 | 1.62 | up | 0 |
|  |  | meta_1396 | Unmapped | 2.20 | 1.79 | up | 0 |
|  |  | meta_1417 | Unmapped | 0.50 | 1.58 | down | 0 |
|  | 72h | meta_1 | Unmapped | 0.33 | 1.49 | down | 0 |
|  |  | meta_6 | Unmapped | 4.39 | 1.48 | up | 0 |
|  |  | meta_10 | 3-Hydroxyisovaleric acid | 0.27 | 1.79 | down | 0 |
|  |  | meta_11 | Ethylenethiourea | 0.47 | 1.29 | down | 0 |
|  |  | meta_13 | Unmapped | 2.10 | 1.73 | up | 0 |
|  |  | meta_15 | L-Serine | 2.40 | 1.77 | up | 15 |
|  |  | meta_31 | Unmapped | 0.48 | 1.28 | down | 0 |
|  |  | meta_38 | L-Threonine | 2.79 | 1.77 | up | 10 |
|  |  | meta_39 | Purine | 5.21 | 1.59 | up | 0 |
|  |  | meta_42 | Unmapped | 0.49 | 1.46 | down | 0 |
|  |  | meta_51 | N-Acetyl-L-alanine | 0.49 | 1.59 | down | 0 |
|  |  | meta_52 | Unmapped | 2.06 | 1.72 | up | 0 |
|  |  | meta_53 | L-Leucine | 3.89 | 1.77 | up | 11 |
|  |  | meta_54 | L-Asparagine | 2.19 | 1.23 | up | 7 |
|  |  | meta_55 | Maleamic acid | 2.98 | 1.69 | up | 1 |
|  |  | meta_57 | D-Aspartic acid | 2.98 | 1.18 | up | 1 |
|  |  | meta_62 | Hypoxanthine | 2.11 | 1.51 | up | 2 |
|  |  | meta_77 | L-Glutamine | 3.04 | 1.80 | up | 17 |
|  |  | meta_81 | Methyl 4-(methylthio)butyrate | 3.33 | 1.78 | up | 0 |
|  |  | meta_82 | L-Methionine | 3.66 | 1.78 | up | 8 |
|  |  | meta_90 | L-Histidine | 2.23 | 1.66 | up | 8 |
|  |  | meta_93 | Orotate | 33.59 | 1.72 | up | 2 |
|  |  | meta_96 | L-dihydroorotate | 13.49 | 1.83 | up | 2 |
|  |  | meta_101 | 2-Oxoadipic acid | 2.03 | 1.67 | up | 6 |
|  |  | meta_103 | D-Ala-D-Ala | 2.46 | 1.18 | up | 0 |
|  |  | meta_107 | Unmapped | 3.56 | 1.38 | up | 0 |
|  |  | meta_111 | Phenylpyruvate | 3.51 | 1.55 | up | 5 |
|  |  | meta_112 | L-Phenylalanine | 3.64 | 1.79 | up | 10 |
|  |  | meta_116 | Trans-3-hydroxy-L-proline | 0.40 | 1.52 | down | 0 |
|  |  | meta_125 | Imetit | 0.39 | 1.29 | down | 0 |
|  |  | meta_126 | Cytosine | 2.94 | 1.35 | up | 2 |
|  |  | meta_130 | L-prolyl-L-glycine | 2.44 | 1.62 | up | 0 |
|  |  | meta_139 | N-carbamoyl-L-aspartate | 3.93 | 1.52 | up | 3 |
|  |  | meta_140 | 5,6,7,8-tetrahydro-2-Naphthoic Acid | 3.16 | 1.51 | up | 0 |
|  |  | meta_145 | Aspirin | 2.63 | 1.50 | up | 1 |
|  |  | meta_148 | L-Tyrosine | 2.89 | 1.79 | up | 19 |
|  |  | meta_152 | Hydroxyphenyllactic acid | 0.31 | 1.77 | down | 0 |
|  |  | meta_153 | Diisopropyl sulfate | 0.35 | 1.33 | down | 0 |
|  |  | meta_165 | 4-Hydroxyphthalide | 2.81 | 1.64 | up | 0 |
|  |  | meta_168 | Glycyl-L-leucine | 4.02 | 1.25 | up | 0 |
|  |  | meta_169 | N6-Acetyl-L-lysine | 2.07 | 1.51 | up | 1 |
|  |  | meta_177 | 1-Isothiocyanato-4-phenylbutane | 4.08 | 1.72 | up | 0 |
|  |  | meta_208 | L-Tryptophan | 4.15 | 1.79 | up | 12 |
|  |  | meta_210 | Pyrogallol-2-O-sulphate | 2.42 | 1.74 | up | 0 |
|  |  | meta_216 | 2,4-Dichloro-3-oxoadipate | 0.47 | 1.43 | down | 0 |
|  |  | meta_219 | D-Ribulose 5-phosphate | 0.18 | 1.80 | down | 7 |
|  |  | meta_220 | Benzyl Benzoate | 3.06 | 1.74 | up | 0 |
|  |  | meta_221 | Omethoate | 0.23 | 1.79 | down | 0 |
|  |  | meta_227 | sn-Glycerol 3-phosphoethanolamine | 2.63 | 1.54 | up | 0 |
|  |  | meta_231 | 5-Hydroxysebacate | 2.98 | 1.20 | up | 0 |
|  |  | meta_234 | 2-Iodophenol | 3.39 | 1.58 | up | 0 |
|  |  | meta_238 | Pseudoecgonine | 2.19 | 1.27 | up | 0 |
|  |  | meta_247 | Phensuximide | 0.39 | 1.49 | down | 0 |
|  |  | meta_248 | Garcinia acid | 0.38 | 1.45 | down | 0 |
|  |  | meta_252 | 3-Indolepropionic acid | 0.43 | 1.17 | down | 0 |
|  |  | meta_265 | Suberylglycine | 3.45 | 1.28 | up | 0 |
|  |  | meta_273 | Unmapped | 2.65 | 1.52 | up | 0 |
|  |  | meta_278 | 1-(2,4,5-Trimethoxyphenyl)-1,2-propanedione | 6.04 | 1.43 | up | 0 |
|  |  | meta_287 | Methasulfocarb | 4.77 | 1.70 | up | 0 |
|  |  | meta_299 | D-Biotin | 0.24 | 1.82 | down | 4 |
|  |  | meta_308 | (E)-2-(2-Furyl)-3-(5-nitro-2-furyl)acrylamide | 3.43 | 1.79 | up | 0 |
|  |  | meta_314 | Deoxyinosine | 2.63 | 1.33 | up | 2 |
|  |  | meta_317 | Unmapped | 0.47 | 1.19 | down | 0 |
|  |  | meta_325 | p-hydroxymexiletine | 0.41 | 1.17 | down | 0 |
|  |  | meta_326 | Dihydrothymine | 4.41 | 1.24 | up | 0 |
|  |  | meta_331 | Pyro-L-glutaminyl-L-glutamine | 2.20 | 1.57 | up | 0 |
|  |  | meta_332 | 3-methylcytidine | 0.48 | 1.75 | down | 0 |
|  |  | meta_339 | Primaquine | 2.56 | 1.69 | up | 0 |
|  |  | meta_341 | Prolyl-Gamma-glutamate | 2.78 | 1.46 | up | 0 |
|  |  | meta_344 | Carbadox | 2.48 | 1.57 | up | 0 |
|  |  | meta_351 | Pemirolast | 2.17 | 1.72 | up | 0 |
|  |  | meta_353 | Tavulin | 3.93 | 1.22 | up | 0 |
|  |  | meta_358 | Inosine | 2.20 | 1.44 | up | 2 |
|  |  | meta_361 | Unmapped | 0.37 | 1.66 | down | 0 |
|  |  | meta_362 | Diallat | 0.38 | 1.47 | down | 0 |
|  |  | meta_377 | D-Glucuronic acid 1-phosphate | 3.06 | 1.54 | up | 0 |
|  |  | meta_378 | Gamma-glutamyl-Gamma-glutamate | 4.77 | 1.66 | up | 0 |
|  |  | meta_394 | 2-Iodophenol | 3.58 | 1.57 | up | 0 |
|  |  | meta_397 | L-Aspartyl-L-phenylalanine | 3.76 | 1.24 | up | 0 |
|  |  | meta_408 | Xanthosine | 0.15 | 1.80 | down | 3 |
|  |  | meta_423 | Methyl 2-(4-isopropyl-4-methyl-5-oxo-2-imidazolin-2-yl)-p-toluate;Methyl 6-(4-isopropyl-4-methyl-5-oxo-2-imidazolin-2-yl)-m-toluate | 4.15 | 1.59 | up | 0 |
|  |  | meta_431 | Phloretin | 2.23 | 1.63 | up | 0 |
|  |  | meta_435 | gamma-L-Glutamyl-L-phenylalanine | 2.74 | 1.25 | up | 0 |
|  |  | meta_437 | 5-Methylcytidine | 2.18 | 1.70 | up | 0 |
|  |  | meta_441 | 2'-O-Methyluridine | 2.28 | 1.80 | up | 0 |
|  |  | meta_454 | N-Acetylgalactosamine 4-sulphate | 0.49 | 1.46 | down | 0 |
|  |  | meta_459 | Unmapped | 0.48 | 1.57 | down | 0 |
|  |  | meta_460 | Malathion monocarboxylic acid | 3.35 | 1.56 | up | 0 |
|  |  | meta_462 | Neuromedin B (1-3) | 6.13 | 1.52 | up | 0 |
|  |  | meta_464 | Eicosapentaenoic Acid | 3.05 | 1.36 | up | 1 |
|  |  | meta_467 | Hesperetin | 4.11 | 1.79 | up | 0 |
|  |  | meta_472 | Arachidonic Acid (peroxide free) | 2.50 | 1.75 | up | 0 |
|  |  | meta_478 | 4-Hydroxy-5-(dihydroxyphenyl)-valeric acid-O-sulphate III | 4.70 | 1.58 | up | 0 |
|  |  | meta_479 | Busulfan | 3.78 | 1.51 | up | 0 |
|  |  | meta_486 | Schradan | 0.20 | 1.67 | down | 0 |
|  |  | meta_488 | N-Acetylneuraminic acid | 0.28 | 1.69 | down | 2 |
|  |  | meta_496 | Lycocernuine | 2.59 | 1.50 | up | 0 |
|  |  | meta_498 | Hippeastrine | 0.14 | 1.83 | down | 0 |
|  |  | meta_501 | (S)-alpha-Terpinyl glucoside | 4.80 | 1.54 | up | 0 |
|  |  | meta_503 | Melilotocarpan A | 2.19 | 1.61 | up | 0 |
|  |  | meta_509 | Adipostatin A | 2.56 | 1.79 | up | 0 |
|  |  | meta_513 | Schradan | 2.54 | 1.33 | up | 0 |
|  |  | meta_520 | Unmapped | 0.14 | 1.50 | down | 0 |
|  |  | meta_523 | N2-(3-Hydroxysuccinoyl)arginine | 0.17 | 1.79 | down | 0 |
|  |  | meta_528 | (4Z,7Z,10Z,13Z,16Z,19Z)-4,7,10,13,1 6,19-Docosahexaenoic acid | 3.52 | 1.36 | up | 0 |
|  |  | meta_532 | Inosine 2',3'-cyclic phosphate | 5.00 | 1.60 | up | 0 |
|  |  | meta_533 | 7Z, 10Z, 13Z, 16Z, 19Z-Docosapentaenoic acid | 3.08 | 1.42 | up | 0 |
|  |  | meta_535 | Fluorescein | 2.66 | 1.78 | up | 0 |
|  |  | meta_536 | Adrenic Acid | 2.16 | 1.66 | up | 1 |
|  |  | meta_537 | L-Thyronine | 4.04 | 1.51 | up | 0 |
|  |  | meta_538 | 2-Methyl-1,4-naphthalenediol bis(dihydrogen phosphate) | 59.87 | 1.47 | up | 0 |
|  |  | meta_539 | Nicotinamide ribotide | 2.47 | 1.75 | up | 0 |
|  |  | meta_542 | Penicillin G | 0.41 | 1.48 | down | 1 |
|  |  | meta_543 | Cappariloside A | 5.10 | 1.67 | up | 0 |
|  |  | meta_545 | Docosatrienoic Acid | 2.93 | 1.67 | up | 0 |
|  |  | meta_551 | Versicolorin A | 36.84 | 1.79 | up | 0 |
|  |  | meta_552 | Salidroside | 2.18 | 1.58 | up | 1 |
|  |  | meta_567 | Zaleplon | 0.14 | 1.43 | down | 0 |
|  |  | meta_590 | Fluorescein | 2.29 | 1.76 | up | 0 |
|  |  | meta_591 | 2-O-p-Coumaroylhydroxycitric acid | 0.40 | 1.70 | down | 0 |
|  |  | meta_595 | 2-Hydroxy-4-(4-methoxyphenyl)-1H-phenalen-1-one | 0.17 | 1.50 | down | 0 |
|  |  | meta_603 | Thenylchlor | 0.29 | 1.49 | down | 0 |
|  |  | meta_604 | Norendoxifen | 3.03 | 1.20 | up | 0 |
|  |  | meta_610 | Pyriminobac-methyl | 2.42 | 1.72 | up | 0 |
|  |  | meta_617 | 9-Aminocamptothecin | 3.29 | 1.72 | up | 0 |
|  |  | meta_622 | Unmapped | 2.53 | 1.51 | up | 0 |
|  |  | meta_623 | Uridine 2',3'-cyclic phosphate | 0.26 | 1.38 | down | 0 |
|  |  | meta_633 | Unmapped | 8.20 | 1.60 | up | 0 |
|  |  | meta_637 | Transfluthrin | 2.90 | 1.78 | up | 0 |
|  |  | meta_639 | Compound III(S) | 3.01 | 1.51 | up | 0 |
|  |  | meta_642 | N-Jasmonoyltyrosine | 3.26 | 1.17 | up | 0 |
|  |  | meta_646 | Prochloraz | 2.25 | 1.75 | up | 0 |
|  |  | meta_652 | Kinetin-9-N-glucoside | 2.34 | 1.57 | up | 0 |
|  |  | meta_657 | Pioglitazone | 0.11 | 1.84 | down | 0 |
|  |  | meta_660 | Salbutamol 4-O-sulfate | 12.51 | 1.85 | up | 0 |
|  |  | meta_667 | Unmapped | 2.77 | 1.61 | up | 0 |
|  |  | meta_672 | Methylsyringin | 4.08 | 1.71 | up | 0 |
|  |  | meta_674 | Cyhexatin | 3.19 | 1.69 | up | 0 |
|  |  | meta_677 | Unmapped | 3.02 | 1.52 | up | 0 |
|  |  | meta_684 | Ajmalicine | 2.02 | 1.27 | up | 1 |
|  |  | meta_686 | Unmapped | 3.58 | 1.49 | up | 0 |
|  |  | meta_690 | Shanzhiside | 2.62 | 1.68 | up | 0 |
|  |  | meta_691 | Unmapped | 0.21 | 1.53 | down | 0 |
|  |  | meta_707 | Unmapped | 5.23 | 1.65 | up | 0 |
|  |  | meta_714 | Uridine 5'-diphosphate | 3.06 | 1.63 | up | 0 |
|  |  | meta_716 | Unmapped | 3.12 | 1.32 | up | 0 |
|  |  | meta_719 | Ancistrocladine | 2.57 | 1.59 | up | 0 |
|  |  | meta_720 | Transfluthrin | 2.13 | 1.72 | up | 0 |
|  |  | meta_724 | Unmapped | 3.56 | 1.34 | up | 0 |
|  |  | meta_731 | Azidocillin | 2.10 | 1.43 | up | 0 |
|  |  | meta_743 | Candoxatrilat | 3.55 | 1.46 | up | 0 |
|  |  | meta_746 | Rumexoside | 3.59 | 1.52 | up | 0 |
|  |  | meta_750 | Benzbromarone | 11.95 | 1.81 | up | 0 |
|  |  | meta_751 | Adifoline | 0.31 | 1.64 | down | 0 |
|  |  | meta_763 | 3-O-Caffeoyl-1-O-methylquinic acid | 2.25 | 1.68 | up | 0 |
|  |  | meta_770 | Unmapped | 0.33 | 1.77 | down | 0 |
|  |  | meta_775 | Fe(III)dicitrate | 3.84 | 1.67 | up | 0 |
|  |  | meta_778 | Phosacetim | 2.92 | 1.40 | up | 0 |
|  |  | meta_785 | Margrapine A | 7.53 | 1.81 | up | 0 |
|  |  | meta_789 | Quinapril | 2.56 | 1.50 | up | 0 |
|  |  | meta_793 | Unmapped | 0.32 | 1.81 | down | 0 |
|  |  | meta_797 | Oxacillin | 2.20 | 1.66 | up | 0 |
|  |  | meta_802 | 3'-Geranyl-2',3,4,4'-tetrahydroxychalcone | 2.32 | 1.70 | up | 0 |
|  |  | meta_806 | CDP-ethanolamine | 2.09 | 1.49 | up | 2 |
|  |  | meta_807 | Mollicellin E | 7.24 | 1.85 | up | 0 |
|  |  | meta_815 | Glycochenodeoxycholate | 0.37 | 1.81 | down | 2 |
|  |  | meta_817 | Unmapped | 2.69 | 1.65 | up | 0 |
|  |  | meta_840 | Unmapped | 2.34 | 1.79 | up | 0 |
|  |  | meta_852 | 2-(a-Hydroxyethyl)thiamine diphosphate | 0.50 | 1.31 | down | 0 |
|  |  | meta_856 | Clofazimine | 0.33 | 1.73 | down | 0 |
|  |  | meta_858 | Sildenafil | 0.49 | 1.76 | down | 0 |
|  |  | meta_916 | Unmapped | 2.51 | 1.73 | up | 0 |
|  |  | meta_919 | Unmapped | 2.15 | 1.36 | up | 0 |
|  |  | meta_922 | Isoglobotriaose | 2.04 | 1.37 | up | 0 |
|  |  | meta_926 | Phosphoadenosine phosphosulfate | 0.29 | 1.28 | down | 0 |
|  |  | meta_927 | Unmapped | 0.44 | 1.59 | down | 0 |
|  |  | meta_932 | Unmapped | 0.20 | 1.30 | down | 0 |
|  |  | meta_937 | Carindone | 0.49 | 1.67 | down | 0 |
|  |  | meta_938 | Unmapped | 0.25 | 1.80 | down | 0 |
|  |  | meta_943 | Unmapped | 2.61 | 1.82 | up | 0 |
|  |  | meta_958 | Unmapped | 2.03 | 1.18 | up | 0 |
|  |  | meta_971 | Unmapped | 0.49 | 1.65 | down | 0 |
|  |  | meta_972 | Unmapped | 0.36 | 1.28 | down | 0 |
|  |  | meta_976 | Hydroxybuprenorphine | 0.14 | 1.30 | down | 0 |
|  |  | meta_978 | Vaccinoside | 3.87 | 1.37 | up | 0 |
|  |  | meta_980 | Corchorosol A | 2.12 | 1.41 | up | 0 |
|  |  | meta_982 | Apramycin | 3.14 | 1.68 | up | 0 |
|  |  | meta_983 | Unmapped | 0.32 | 1.28 | down | 0 |
|  |  | meta_985 | Unmapped | 0.24 | 1.82 | down | 0 |
|  |  | meta_999 | ;Penitrem D | 0.37 | 1.54 | down | 0 |
|  |  | meta_1008 | Unmapped | 2.51 | 1.80 | up | 0 |
|  |  | meta_1011 | DMG-MINO | 2.41 | 1.64 | up | 0 |
|  |  | meta_1015 | Albanol B | 2.27 | 1.26 | up | 0 |
|  |  | meta_1019 | Unmapped | 0.22 | 1.63 | down | 0 |
|  |  | meta_1025 | 2-O-[2-O-(alpha-D-Mannopyranosyl)-alpha-D-glucopyranosyl]-3-phospho-D-glycerate | 2.49 | 1.56 | up | 0 |
|  |  | meta_1028 | Isotheaflavin | 2.05 | 1.50 | up | 0 |
|  |  | meta_1032 | Unmapped | 2.08 | 1.40 | up | 0 |
|  |  | meta_1034 | Unmapped | 2.02 | 1.60 | up | 0 |
|  |  | meta_1058 | dTDP-D-mycarose | 2.09 | 1.50 | up | 0 |
|  |  | meta_1068 | Uridine diphosphate glucose | 2.63 | 1.38 | up | 0 |
|  |  | meta_1087 | Unmapped | 2.14 | 1.37 | up | 0 |
|  |  | meta_1092 | Pelargonidin 3-O-3'',6''-O-dimalonylglucoside | 2.06 | 1.29 | up | 0 |
|  |  | meta_1099 | Unmapped | 2.94 | 1.39 | up | 0 |
|  |  | meta_1101 | Glutathione disulfide | 2.71 | 1.70 | up | 2 |
|  |  | meta_1107 | Dimoracin | 5.01 | 1.77 | up | 0 |
|  |  | meta_1113 | Lanceotoxin A | 3.40 | 1.56 | up | 0 |
|  |  | meta_1115 | Isotetrandrine | 3.01 | 1.69 | up | 0 |
|  |  | meta_1117 | Cholesteryl palmitate | 2.31 | 1.73 | up | 0 |
|  |  | meta_1120 | Geranyl diphosphate | 2.68 | 1.65 | up | 3 |
|  |  | meta_1154 | N-Lignoceroylsphingosine | 2.52 | 1.65 | up | 0 |
|  |  | meta_1163 | Rifamycin W | 0.47 | 1.72 | down | 0 |
|  |  | meta_1168 | Streptomycin 6-phosphate | 5.13 | 1.73 | up | 0 |
|  |  | meta_1212 | Congo red | 2.05 | 1.59 | up | 0 |
|  |  | meta_1215 | Adouetine Z | 3.74 | 1.66 | up | 0 |
|  |  | meta_1219 | Unmapped | 0.43 | 1.15 | down | 0 |
|  |  | meta_1225 | N-Lignoceroylsphingosine | 2.04 | 1.64 | up | 0 |
|  |  | meta_1230 | PE(P-18:0/14:0) | 0.47 | 1.39 | down | 0 |
|  |  | meta_1239 | PE(P-18:1(9Z)/16:1(9Z)) | 2.65 | 1.27 | up | 0 |
|  |  | meta_1245 | Unmapped | 0.30 | 1.78 | down | 0 |
|  |  | meta_1265 | PE(P-18:1(11Z)/20:5(5Z,8Z,11Z,14Z,17Z)) | 3.71 | 1.31 | up | 0 |
|  |  | meta_1271 | Unmapped | 2.26 | 1.72 | up | 0 |
|  |  | meta_1275 | Unmapped | 0.14 | 1.80 | down | 0 |
|  |  | meta_1291 | PC(20:3(5Z,8Z,11Z)/15:0) | 2.23 | 1.57 | up | 0 |
|  |  | meta_1296 | PE(22:6(4Z,7Z,10Z,13Z,16Z,19Z)/P-18:1(11Z)) | 3.91 | 1.42 | up | 0 |
|  |  | meta_1300 | PC(18:2(9Z,12Z)/18:3(9Z,12Z,15Z)) | 0.50 | 1.43 | down | 0 |
|  |  | meta_1302 | PE(18:3(6Z,9Z,12Z)/22:5(4Z,7Z,10Z,13Z,16Z)) | 2.33 | 1.09 | up | 0 |
|  |  | meta_1319 | PC(o-16:0/22:0) | 3.61 | 1.41 | up | 0 |
|  |  | meta_1327 | Quinquenoside F1 | 2.46 | 1.31 | up | 0 |
|  |  | meta_1332 | Punigluconin | 2.05 | 1.73 | up | 0 |
|  |  | meta_1335 | PC(o-16:0/22:0) | 2.58 | 1.38 | up | 0 |
|  |  | meta_1348 | PC(o-16:0/22:0) | 2.53 | 1.35 | up | 0 |
|  |  | meta_1350 | PE(22:2(13Z,16Z)/22:6(4Z,7Z,10Z,13Z,16Z,19Z)) | 2.07 | 1.37 | up | 0 |
|  |  | meta_1351 | PG(18:0/22:4(7Z,10Z,13Z,16Z) | 2.06 | 1.55 | up | 0 |
|  |  | meta_1355 | PE(24:1(15Z)/20:5(5Z,8Z,11Z,14Z,17Z)) | 2.24 | 1.24 | up | 0 |
|  |  | meta_1356 | Unmapped | 2.17 | 1.44 | up | 0 |
|  |  | meta_1365 | Unmapped | 2.89 | 1.23 | up | 0 |
|  |  | meta_1366 | Unmapped | 2.82 | 1.16 | up | 0 |
|  |  | meta_1376 | Elatoside E | 2.16 | 1.21 | up | 0 |
|  |  | meta_1377 | PI(20:3(8Z,11Z,14Z)/18:2(9Z,12Z)) | 3.12 | 1.77 | up | 0 |
|  |  | meta_1381 | Unmapped | 2.55 | 1.37 | up | 0 |
|  |  | meta_1388 | Bacteriochlorophyll b | 2.16 | 1.26 | up | 0 |
|  |  | meta_1410 | Unmapped | 2.51 | 1.39 | up | 0 |
|  |  | meta_1417 | Unmapped | 0.48 | 1.58 | down | 0 |
| neg | 48h | meta_3 | Dimethylformamide | 2.28 | 1.71 | up | 0 |
|  |  | meta_7 | Unmapped | 2.12 | 1.88 | up | 0 |
|  |  | meta_12 | Unmapped | 2.40 | 1.71 | up | 0 |
|  |  | meta_17 | L-Serine | 2.33 | 1.80 | up | 15 |
|  |  | meta_22 | Hypotaurine | 2.47 | 1.63 | up | 2 |
|  |  | meta_34 | L-Threonine | 2.83 | 1.83 | up | 10 |
|  |  | meta_36 | Tyramine | 2.49 | 1.75 | up | 3 |
|  |  | meta_38 | Purine | 3.93 | 1.63 | up | 0 |
|  |  | meta_41 | Picolinic acid | 0.43 | 1.55 | down | 2 |
|  |  | meta_51 | L-Leucine | 2.28 | 1.85 | up | 11 |
|  |  | meta_53 | L-Asparagine | 2.08 | 1.86 | up | 7 |
|  |  | meta_56 | D-Aspartic acid | 3.56 | 1.77 | up | 1 |
|  |  | meta_59 | Adenine | 0.48 | 1.15 | down | 2 |
|  |  | meta_78 | L-Glutamine | 3.12 | 1.58 | up | 17 |
|  |  | meta_93 | Lavandulol | 2.84 | 1.22 | up | 0 |
|  |  | meta_99 | Tryptamine | 2.06 | 1.31 | up | 3 |
|  |  | meta_109 | L-Phenylalanine | 2.38 | 1.75 | up | 10 |
|  |  | meta_114 | 6-Hydroxydopamine | 2.15 | 1.55 | up | 0 |
|  |  | meta_120 | Pro-Gly | 2.05 | 1.78 | up | 0 |
|  |  | meta_129 | Tolazoline | 0.43 | 1.47 | down | 0 |
|  |  | meta_133 | L-Tyrosine | 2.01 | 1.82 | up | 19 |
|  |  | meta_136 | 4-Hydroxybenzaldehyde | 2.01 | 1.70 | up | 1 |
|  |  | meta_146 | DL-Indole-3-lactic acid | 2.35 | 1.83 | up | 0 |
|  |  | meta_179 | N-Acetyl-D-glucosamine | 2.23 | 1.49 | up | 3 |
|  |  | meta_181 | L-Tryptophan | 2.31 | 1.82 | up | 12 |
|  |  | meta_186 | Methylthiouracil | 2.37 | 1.73 | up | 0 |
|  |  | meta_216 | N-Acetylmannosamine | 2.37 | 1.59 | up | 0 |
|  |  | meta_217 | Unmapped | 3.12 | 1.82 | up | 0 |
|  |  | meta_251 | Meteloidine | 0.35 | 1.15 | down | 0 |
|  |  | meta_252 | Tyrosyl-Glycine | 2.46 | 1.53 | up | 0 |
|  |  | meta_253 | Tyr-Gly | 2.51 | 1.78 | up | 0 |
|  |  | meta_261 | L-2-Amino-3-(oxalylamino)propanoic acid | 7.53 | 1.85 | up | 0 |
|  |  | meta_262 | D-Mannose | 2.52 | 1.37 | up | 6 |
|  |  | meta_270 | Monoethylglycylxylidide | 0.49 | 1.66 | down | 0 |
|  |  | meta_271 | Pro-Glu | 2.31 | 1.30 | up | 0 |
|  |  | meta_275 | 2-Methylbutyroylcarnitine | 0.13 | 1.81 | down | 0 |
|  |  | meta_280 | 2,6-Diamino-4-hydroxy-5-N-methylformamidopyrimidine | 0.38 | 1.40 | down | 0 |
|  |  | meta_319 | 1-Amino-3-hydroxymethyl-5-methyl-adamantane | 4.22 | 1.53 | up | 0 |
|  |  | meta_324 | Primaquine | 2.40 | 1.81 | up | 0 |
|  |  | meta_325 | Lysyl-Leucine | 0.39 | 1.33 | down | 0 |
|  |  | meta_334 | Lys-Pro | 0.41 | 1.33 | down | 0 |
|  |  | meta_343 | 1-Chloro-2,2-bis(4'-chlorophenyl)ethylene | 6.44 | 1.87 | up | 0 |
|  |  | meta_359 | Pro-Asn | 2.08 | 1.43 | up | 0 |
|  |  | meta_379 | Phenylalanyl-Isoleucine | 2.15 | 1.55 | up | 0 |
|  |  | meta_391 | 1-Chloro-2,2-bis(4'-chlorophenyl)ethylene | 2.58 | 1.75 | up | 0 |
|  |  | meta_401 | Zolmitriptan | 2.50 | 1.45 | up | 0 |
|  |  | meta_403 | Unmapped | 6.13 | 1.88 | up | 0 |
|  |  | meta_425 | Phe-Glu | 2.52 | 1.35 | up | 0 |
|  |  | meta_430 | 1-methylguanosine | 2.09 | 1.55 | up | 0 |
|  |  | meta_438 | Unmapped | 0.39 | 1.15 | down | 0 |
|  |  | meta_459 | Xanthosine | 0.46 | 1.40 | down | 3 |
|  |  | meta_475 | Flusilazole | 0.04 | 1.74 | down | 0 |
|  |  | meta_481 | Arg-Thr | 4.85 | 1.75 | up | 0 |
|  |  | meta_502 | Norelgestromin | 2.12 | 1.48 | up | 0 |
|  |  | meta_503 | Oxprenolol | 2.25 | 1.55 | up | 0 |
|  |  | meta_520 | Triflupromazine | 2.83 | 1.72 | up | 0 |
|  |  | meta_525 | D-Ribose | 2.47 | 1.78 | up | 2 |
|  |  | meta_543 | Adenosine monophosphate | 0.48 | 1.41 | down | 0 |
|  |  | meta_545 | Butote | 6.36 | 1.85 | up | 0 |
|  |  | meta_589 | (4Z,7Z,10Z,13Z,16Z,19Z)-4,7,10,13,1 6,19-Docosahexaenoic acid | 4.39 | 1.39 | up | 0 |
|  |  | meta_597 | 1,2-O-Diacetylzephyranthine | 2.46 | 1.59 | up | 0 |
|  |  | meta_598 | Lys-Trp | 2.05 | 1.69 | up | 0 |
|  |  | meta_606 | Cyphenothrin | 0.49 | 1.46 | down | 0 |
|  |  | meta_618 | Persicaxanthin | 2.36 | 1.69 | up | 0 |
|  |  | meta_623 | Neolinustatin | 2.01 | 1.16 | up | 0 |
|  |  | meta_653 | 4-Hydroxyphenylacetonitrile triacetylrhamnoside | 2.49 | 1.63 | up | 0 |
|  |  | meta_671 | Tryprostatin A | 2.22 | 1.73 | up | 0 |
|  |  | meta_674 | Pro-Ser | 3.46 | 1.45 | up | 0 |
|  |  | meta_690 | Abiraterone sulfate | 4.18 | 1.82 | up | 0 |
|  |  | meta_706 | N-Arachidonyl dopamine | 0.44 | 1.62 | down | 0 |
|  |  | meta_718 | CDP-ethanolamine | 2.31 | 1.78 | up | 2 |
|  |  | meta_721 | Unmapped | 0.44 | 1.49 | down | 0 |
|  |  | meta_740 | Retinyl beta-glucuronide | 2.13 | 1.72 | up | 0 |
|  |  | meta_745 | PSF-A | 2.87 | 1.53 | up | 0 |
|  |  | meta_750 | 1-Myristoyl-sn-glycero-3-phosphocholine | 0.32 | 1.61 | down | 0 |
|  |  | meta_756 | Unmapped | 2.51 | 1.70 | up | 0 |
|  |  | meta_766 | Unmapped | 4.11 | 1.61 | up | 0 |
|  |  | meta_770 | 1-O-Hexadecyl-lyso-sn-glycero-3-phosphocholine | 0.31 | 1.65 | down | 0 |
|  |  | meta_794 | LysoPE(18:2(9Z,12Z)/0:0) | 2.50 | 1.57 | up | 0 |
|  |  | meta_808 | 1-O-Octadecyl-sn-glyceryl-3-phosphorylcholine | 0.41 | 1.74 | down | 0 |
|  |  | meta_816 | Nb-trans-Feruloylserotonin glucoside | 2.57 | 1.43 | up | 0 |
|  |  | meta_831 | LysoPE(18:0/0:0) | 2.66 | 1.65 | up | 0 |
|  |  | meta_863 | Cohibin B | 0.45 | 1.71 | down | 0 |
|  |  | meta_873 | Ile-Phe | 2.20 | 1.58 | up | 0 |
|  |  | meta_877 | Nb-trans-Feruloylserotonin glucoside | 2.23 | 1.61 | up | 0 |
|  |  | meta_886 | 1-Stearoyl-sn-glycerol 3-phosphocholine | 2.02 | 1.55 | up | 0 |
|  |  | meta_898 | Unmapped | 10.19 | 1.89 | up | 0 |
|  |  | meta_917 | Unmapped | 3.94 | 1.70 | up | 0 |
|  |  | meta_921 | Unmapped | 5.20 | 1.90 | up | 0 |
|  |  | meta_923 | Unmapped | 2.29 | 1.83 | up | 0 |
|  |  | meta_927 | 10-Acetoxyoleuropein | 2.73 | 1.69 | up | 0 |
|  |  | meta_933 | Lymecycline | 2.75 | 1.44 | up | 0 |
|  |  | meta_962 | Quercetin 7-glucuronide 3-rhamnoside | 2.59 | 1.69 | up | 0 |
|  |  | meta_965 | Kuwanon L | 2.94 | 1.66 | up | 0 |
|  |  | meta_991 | Unmapped | 3.75 | 1.78 | up | 0 |
|  |  | meta_992 | 6''-Malonylapiin | 2.84 | 1.53 | up | 0 |
|  |  | meta_1005 | Unmapped | 2.16 | 1.61 | up | 0 |
|  |  | meta_1027 | Unmapped | 0.40 | 1.57 | down | 0 |
|  |  | meta_1032 | Norbadione A | 0.30 | 1.88 | down | 0 |
|  |  | meta_1060 | DG(22:6(4Z,7Z,10Z,13Z,16Z,19Z)/20:5(5Z,8Z,11Z,14Z,17Z)/0:0) | 0.39 | 1.87 | down | 0 |
|  |  | meta_1109 | PC(18:1(11Z)/18:3(9Z,12Z,15Z)) | 2.01 | 1.62 | up | 0 |
|  |  | meta_1111 | PC(o-16:1(9Z)/20:4(8Z,11Z,14Z,17Z)) | 3.99 | 1.26 | up | 0 |
|  |  | meta_1113 | Torvoside E | 4.31 | 1.75 | up | 0 |
|  |  | meta_1115 | Unmapped | 2.19 | 1.92 | up | 0 |
|  |  | meta_1126 | Unmapped | 3.22 | 1.58 | up | 0 |
|  |  | meta_1127 | Phyllanthusol B | 2.01 | 1.65 | up | 0 |
|  |  | meta_1139 | Phyllanthusol B | 2.03 | 1.50 | up | 0 |
|  |  | meta_1143 | PC(22:5(7Z,10Z,13Z,16Z,19Z)/P-18:1(11Z)) | 2.11 | 1.67 | up | 0 |
|  |  | meta_1144 | PC(22:5(7Z,10Z,13Z,16Z,19Z)/P-18:1(11Z)) | 3.25 | 1.71 | up | 0 |
|  |  | meta_1148 | Lactosylceramide (d18:1/12:0) | 2.45 | 1.64 | up | 0 |
|  |  | meta_1149 | 1,2-dioleoyl-sn-glycero-3-phosphatidylcholine | 2.52 | 1.84 | up | 0 |
|  |  | meta_1151 | 1-Stearoyl-2-oleoyl-sn-glycerol 3-phosphocholine | 2.37 | 1.65 | up | 0 |
|  |  | meta_1154 | PC(18:2(9Z,12Z)/20:1(11Z)) | 3.96 | 1.66 | up | 0 |
|  |  | meta_1159 | Unmapped | 2.13 | 1.75 | up | 0 |
|  |  | meta_1162 | PG(18:3(6Z,9Z,12Z)/22:4(7Z,10Z,13Z,16Z)) | 2.67 | 1.77 | up | 0 |
|  |  | meta_1170 | Isobutyryl-CoA | 2.87 | 1.46 | up | 0 |
|  |  | meta_1176 | PG(18:1(9Z)/22:5(7Z,10Z,13Z,16Z,19Z)) | 2.85 | 1.60 | up | 0 |
|  |  | meta_1181 | Unmapped | 3.83 | 1.89 | up | 0 |
|  |  | meta_1192 | PI(20:3(8Z,11Z,14Z)/18:2(9Z,12Z)) | 2.35 | 1.48 | up | 0 |
|  |  | meta_1212 | Unmapped | 2.20 | 1.28 | up | 0 |
|  |  | meta_1215 | Unmapped | 2.04 | 1.50 | up | 0 |
|  | 72h | meta_2 | Pyrrolidine | 2.82 | 1.76 | up | 0 |
|  |  | meta_3 | Dimethylformamide | 2.40 | 1.74 | up | 0 |
|  |  | meta_6 | gamma-Aminobutryic acid | 0.36 | 1.61 | down | 0 |
|  |  | meta_7 | Unmapped | 2.98 | 1.78 | up | 0 |
|  |  | meta_12 | Unmapped | 2.81 | 1.70 | up | 0 |
|  |  | meta_17 | L-Serine | 2.54 | 1.70 | up | 15 |
|  |  | meta_22 | Hypotaurine | 2.32 | 1.29 | up | 2 |
|  |  | meta_32 | Phenylacetic acid | 2.06 | 1.77 | up | 2 |
|  |  | meta_34 | L-Threonine | 3.06 | 1.69 | up | 10 |
|  |  | meta_35 | Acetoacetic acid | 2.28 | 1.73 | up | 5 |
|  |  | meta_36 | Tyramine | 2.43 | 1.66 | up | 3 |
|  |  | meta_38 | Purine | 7.18 | 1.57 | up | 0 |
|  |  | meta_41 | Picolinic acid | 0.16 | 1.80 | down | 2 |
|  |  | meta_46 | Diethanolamine | 0.43 | 1.27 | down | 1 |
|  |  | meta_49 | Atrolactic acid | 2.21 | 1.75 | up | 2 |
|  |  | meta_51 | L-Leucine | 2.77 | 1.79 | up | 11 |
|  |  | meta_56 | D-Aspartic acid | 2.31 | 1.44 | up | 1 |
|  |  | meta_66 | Gaboxadol | 0.47 | 1.61 | down | 0 |
|  |  | meta_68 | Taurine | 2.37 | 1.30 | up | 6 |
|  |  | meta_77 | Coumarin | 2.13 | 1.80 | up | 0 |
|  |  | meta_78 | L-Glutamine | 3.25 | 1.77 | up | 16 |
|  |  | meta_82 | trans-cinnamate | 2.26 | 1.80 | up | 3 |
|  |  | meta_83 | L-Methionine | 2.63 | 1.71 | up | 8 |
|  |  | meta_91 | D-Ornithine | 0.41 | 1.24 | down | 2 |
|  |  | meta_96 | D-Glucuronate | 2.42 | 1.76 | up | 5 |
|  |  | meta_98 | Cyclohexylamine | 0.42 | 1.69 | down | 0 |
|  |  | meta_99 | Tryptamine | 2.95 | 1.40 | up | 3 |
|  |  | meta_102 | L-Carnitine | 0.49 | 1.67 | down | 1 |
|  |  | meta_103 | Enol-phenylpyruvate | 2.62 | 1.76 | up | 0 |
|  |  | meta_109 | L-Phenylalanine | 2.57 | 1.68 | up | 10 |
|  |  | meta_110 | Unmapped | 0.45 | 1.44 | down | 0 |
|  |  | meta_116 | L-Pyroglutamic acid | 0.36 | 1.66 | down | 1 |
|  |  | meta_120 | Pro-Gly | 2.89 | 1.63 | up | 0 |
|  |  | meta_123 | Allopurinol | 2.07 | 1.65 | up | 0 |
|  |  | meta_133 | L-Tyrosine | 2.65 | 1.76 | up | 19 |
|  |  | meta_139 | 4-Amino-2-hydroxylamino-6-nitrotoluene | 0.42 | 1.35 | down | 0 |
|  |  | meta_142 | Unmapped | 0.20 | 1.73 | down | 0 |
|  |  | meta_146 | DL-Indole-3-lactic acid | 3.37 | 1.76 | up | 0 |
|  |  | meta_148 | alpha-Guanidinoglutaric Acid | 2.52 | 1.74 | up | 0 |
|  |  | meta_160 | Ornithine | 0.32 | 1.36 | down | 2 |
|  |  | meta_162 | 3-Hydroxy-2-methylpyridine-4,5-dicarboxylate | 0.48 | 1.13 | down | 0 |
|  |  | meta_176 | Benzoyl phosphate | 0.36 | 1.38 | down | 0 |
|  |  | meta_178 | NG,NG-dimethyl-L-arginine | 2.05 | 1.60 | up | 0 |
|  |  | meta_181 | L-Tryptophan | 3.19 | 1.78 | up | 12 |
|  |  | meta_185 | Dihydrolipoate (dihydrolipoic acid) | 0.35 | 1.59 | down | 0 |
|  |  | meta_186 | Methylthiouracil | 2.12 | 1.77 | up | 0 |
|  |  | meta_200 | Sodium glucurote | 3.24 | 1.65 | up | 0 |
|  |  | meta_204 | 7-Aminomethyl-7-deazaguanine | 0.42 | 1.58 | down | 0 |
|  |  | meta_217 | Unmapped | 3.60 | 1.80 | up | 0 |
|  |  | meta_226 | 3-Nitrotyrosine | 0.04 | 1.45 | down | 0 |
|  |  | meta_236 | Octhilinone | 8.03 | 1.55 | up | 0 |
|  |  | meta_244 | Unmapped | 0.29 | 1.22 | down | 0 |
|  |  | meta_251 | Meteloidine | 0.44 | 1.14 | down | 0 |
|  |  | meta_252 | Tyrosyl-Glycine | 4.03 | 1.37 | up | 0 |
|  |  | meta_253 | Tyr-Gly | 2.53 | 1.39 | up | 0 |
|  |  | meta_261 | L-2-Amino-3-(oxalylamino)propanoic acid | 3.63 | 1.48 | up | 0 |
|  |  | meta_271 | Pro-Glu | 2.24 | 1.20 | up | 0 |
|  |  | meta_275 | 2-Methylbutyroylcarnitine | 0.06 | 1.81 | down | 0 |
|  |  | meta_280 | 2,6-Diamino-4-hydroxy-5-N-methylformamidopyrimidine | 0.35 | 1.70 | down | 0 |
|  |  | meta_307 | Meteloidine | 0.37 | 1.29 | down | 0 |
|  |  | meta_316 | Pro-Thr | 2.31 | 1.50 | up | 0 |
|  |  | meta_319 | 1-Amino-3-hydroxymethyl-5-methyl-adamantane | 6.12 | 1.55 | up | 0 |
|  |  | meta_324 | Primaquine | 3.09 | 1.69 | up | 0 |
|  |  | meta_331 | Glycerol tripropanoate | 3.63 | 1.57 | up | 0 |
|  |  | meta_343 | 1-Chloro-2,2-bis(4'-chlorophenyl)ethylene | 3.20 | 1.51 | up | 0 |
|  |  | meta_369 | Lysyl-Glutamate | 5.13 | 1.54 | up | 0 |
|  |  | meta_370 | Viloxazine | 0.31 | 1.35 | down | 0 |
|  |  | meta_371 | Thr-Asp | 2.41 | 1.46 | up | 0 |
|  |  | meta_374 | Hydroxyhexanoycarnitine | 0.40 | 1.83 | down | 0 |
|  |  | meta_380 | Pyro-L-glutaminyl-L-glutamine | 2.18 | 1.60 | up | 0 |
|  |  | meta_403 | Unmapped | 10.80 | 1.66 | up | 0 |
|  |  | meta_406 | Arginyl-Asparagine | 4.11 | 1.57 | up | 0 |
|  |  | meta_409 | Met-Val | 2.66 | 1.57 | up | 0 |
|  |  | meta_423 | Pyridine N-oxide glucuronide | 2.40 | 1.76 | up | 0 |
|  |  | meta_425 | Phe-Glu | 2.56 | 1.36 | up | 0 |
|  |  | meta_437 | Sphingosine | 2.57 | 1.82 | up | 4 |
|  |  | meta_438 | Unmapped | 0.37 | 1.29 | down | 0 |
|  |  | meta_441 | Promethazine | 2.68 | 1.39 | up | 0 |
|  |  | meta_445 | Moprolol | 3.84 | 1.49 | up | 0 |
|  |  | meta_459 | Xanthosine | 0.19 | 1.67 | down | 3 |
|  |  | meta_463 | Tyr-Gln | 0.11 | 1.60 | down | 0 |
|  |  | meta_465 | Murrayacinine | 2.43 | 1.38 | up | 0 |
|  |  | meta_466 | Zanthosimuline | 2.35 | 1.47 | up | 0 |
|  |  | meta_475 | Flusilazole | 0.04 | 1.78 | down | 0 |
|  |  | meta_481 | Arg-Thr | 4.56 | 1.41 | up | 0 |
|  |  | meta_488 | Tryptophyl-Aspartate | 2.04 | 1.47 | up | 0 |
|  |  | meta_498 | Unmapped | 0.32 | 1.20 | down | 0 |
|  |  | meta_502 | Norelgestromin | 3.33 | 1.43 | up | 0 |
|  |  | meta_503 | Oxprenolol | 3.90 | 1.52 | up | 0 |
|  |  | meta_505 | Inosine 2',3'-cyclic phosphate | 3.99 | 1.58 | up | 0 |
|  |  | meta_506 | N1,N12-Diacetylspermine | 4.37 | 1.55 | up | 0 |
|  |  | meta_518 | Benomyl | 0.37 | 1.56 | down | 0 |
|  |  | meta_519 | Cappariloside A | 2.71 | 1.78 | up | 0 |
|  |  | meta_520 | Triflupromazine | 3.16 | 1.74 | up | 0 |
|  |  | meta_543 | Adenosine monophosphate | 0.44 | 1.57 | down | 0 |
|  |  | meta_545 | Butote | 3.60 | 1.57 | up | 0 |
|  |  | meta_551 | Penicilloic acid | 0.49 | 1.60 | down | 0 |
|  |  | meta_552 | Trimethobenzamide | 2.12 | 1.29 | up | 0 |
|  |  | meta_560 | Ile-Tyr | 2.07 | 1.55 | up | 0 |
|  |  | meta_574 | Ethoxysulfuron | 0.49 | 1.36 | down | 0 |
|  |  | meta_582 | Unmapped | 0.40 | 1.20 | down | 0 |
|  |  | meta_589 | (4Z,7Z,10Z,13Z,16Z,19Z)-4,7,10,13,1 6,19-Docosahexaenoic acid | 6.73 | 1.52 | up | 0 |
|  |  | meta_597 | 1,2-O-Diacetylzephyranthine | 2.92 | 1.53 | up | 0 |
|  |  | meta_601 | Indoleacrylic acid | 0.15 | 1.71 | down | 0 |
|  |  | meta_638 | (3beta,22E,24R)-Ergosta-4,6,8(14),22-tetraen-3-ol | 0.40 | 1.12 | down | 0 |
|  |  | meta_643 | L-Palmitoylcarnitine | 2.06 | 1.25 | up | 2 |
|  |  | meta_653 | 4-Hydroxyphenylacetonitrile triacetylrhamnoside | 3.16 | 1.22 | up | 0 |
|  |  | meta_671 | Tryprostatin A | 3.50 | 1.61 | up | 0 |
|  |  | meta_674 | Pro-Ser | 3.25 | 1.42 | up | 0 |
|  |  | meta_682 | Bumetanide | 0.29 | 1.48 | down | 0 |
|  |  | meta_690 | Abiraterone sulfate | 5.75 | 1.65 | up | 0 |
|  |  | meta_697 | Buclizine | 2.56 | 1.22 | up | 0 |
|  |  | meta_706 | N-Arachidonyl dopamine | 0.34 | 1.72 | down | 0 |
|  |  | meta_721 | Unmapped | 0.37 | 1.50 | down | 0 |
|  |  | meta_729 | Unmapped | 0.49 | 1.27 | down | 0 |
|  |  | meta_740 | Retinyl beta-glucuronide | 2.41 | 1.67 | up | 0 |
|  |  | meta_755 | lamotrigine-2-N-glucuronide | 0.46 | 1.18 | down | 0 |
|  |  | meta_766 | Unmapped | 2.86 | 1.34 | up | 0 |
|  |  | meta_770 | 1-O-Hexadecyl-lyso-sn-glycero-3-phosphocholine | 0.11 | 1.76 | down | 0 |
|  |  | meta_773 | Unmapped | 0.49 | 1.11 | down | 0 |
|  |  | meta_781 | Unmapped | 4.93 | 1.60 | up | 0 |
|  |  | meta_784 | Dihydroneopterin triphosphate | 2.35 | 1.64 | up | 0 |
|  |  | meta_795 | Unmapped | 2.17 | 1.67 | up | 0 |
|  |  | meta_799 | Unmapped | 0.48 | 1.35 | down | 0 |
|  |  | meta_808 | 1-O-Octadecyl-sn-glyceryl-3-phosphorylcholine | 0.18 | 1.79 | down | 0 |
|  |  | meta_816 | Nb-trans-Feruloylserotonin glucoside | 3.81 | 1.40 | up | 0 |
|  |  | meta_818 | Palmidin B | 2.20 | 1.49 | up | 0 |
|  |  | meta_846 | Nb-trans-Feruloylserotonin glucoside | 2.43 | 1.53 | up | 0 |
|  |  | meta_851 | Apramycin | 2.78 | 1.72 | up | 0 |
|  |  | meta_865 | Unmapped | 0.34 | 1.73 | down | 0 |
|  |  | meta_869 | Nb-trans-Feruloylserotonin glucoside | 2.10 | 1.28 | up | 0 |
|  |  | meta_873 | Ile-Phe | 2.16 | 1.27 | up | 0 |
|  |  | meta_877 | Nb-trans-Feruloylserotonin glucoside | 2.02 | 1.20 | up | 0 |
|  |  | meta_883 | Isotheaflavin | 2.11 | 1.46 | up | 0 |
|  |  | meta_898 | Unmapped | 8.82 | 1.77 | up | 0 |
|  |  | meta_903 | Unmapped | 2.47 | 1.69 | up | 0 |
|  |  | meta_907 | Streptomycin | 3.58 | 1.74 | up | 0 |
|  |  | meta_916 | Isotheaflavin | 2.07 | 1.60 | up | 0 |
|  |  | meta_917 | Unmapped | 3.67 | 1.56 | up | 0 |
|  |  | meta_918 | Unmapped | 2.01 | 1.62 | up | 0 |
|  |  | meta_921 | Unmapped | 5.13 | 1.80 | up | 0 |
|  |  | meta_923 | Unmapped | 2.97 | 1.74 | up | 0 |
|  |  | meta_943 | Unmapped | 2.03 | 1.47 | up | 0 |
|  |  | meta_950 | Streptomycin | 2.46 | 1.72 | up | 0 |
|  |  | meta_962 | Quercetin 7-glucuronide 3-rhamnoside | 2.03 | 1.54 | up | 0 |
|  |  | meta_963 | CE(16:0) | 2.29 | 1.68 | up | 0 |
|  |  | meta_991 | Unmapped | 2.80 | 1.53 | up | 0 |
|  |  | meta_994 | Unmapped | 0.37 | 1.79 | down | 0 |
|  |  | meta_1005 | Unmapped | 4.62 | 1.60 | up | 0 |
|  |  | meta_1007 | Neocarzinostatin chromophore | 2.44 | 1.70 | up | 0 |
|  |  | meta_1008 | Unmapped | 2.25 | 1.67 | up | 0 |
|  |  | meta_1027 | Unmapped | 0.20 | 1.72 | down | 0 |
|  |  | meta_1028 | Unmapped | 0.33 | 1.77 | down | 0 |
|  |  | meta_1032 | Norbadione A | 0.45 | 1.71 | down | 0 |
|  |  | meta_1065 | Unmapped | 0.49 | 1.55 | down | 0 |
|  |  | meta_1094 | Unmapped | 0.50 | 1.31 | down | 0 |
|  |  | meta_1096 | Unmapped | 0.39 | 1.25 | down | 0 |
|  |  | meta_1098 | Unmapped | 2.26 | 1.64 | up | 0 |
|  |  | meta_1112 | PC(20:3(5Z,8Z,11Z)/15:0) | 2.49 | 1.67 | up | 0 |
|  |  | meta_1113 | Torvoside E | 3.84 | 1.60 | up | 0 |
|  |  | meta_1115 | Unmapped | 2.18 | 1.63 | up | 0 |
|  |  | meta_1127 | Phyllanthusol B | 3.48 | 1.44 | up | 0 |
|  |  | meta_1138 | PC(16:1(9Z)/22:5(4Z,7Z,10Z,13Z,16Z)) | 2.33 | 1.56 | up | 0 |
|  |  | meta_1139 | Phyllanthusol B | 2.42 | 1.22 | up | 0 |
|  |  | meta_1154 | PC(18:2(9Z,12Z)/20:1(11Z)) | 2.33 | 1.35 | up | 0 |
|  |  | meta_1160 | TG(14:1(9Z)/20:3n6/o-18:0) | 2.16 | 1.66 | up | 0 |
|  |  | meta_1161 | Unmapped | 2.16 | 1.70 | up | 0 |
|  |  | meta_1162 | PG(18:3(6Z,9Z,12Z)/22:4(7Z,10Z,13Z,16Z)) | 2.03 | 1.38 | up | 0 |
|  |  | meta_1167 | Malomoyl-CoA | 2.72 | 1.67 | up | 0 |
|  |  | meta_1170 | Isobutyryl-CoA | 2.51 | 1.27 | up | 0 |
|  |  | meta_1181 | Unmapped | 3.69 | 1.77 | up | 0 |
|  |  | meta_1223 | Unmapped | 0.50 | 1.69 | down | 0 |
|  |  | meta_1233 | Condurango glycoside A | 2.98 | 1.47 | up | 0 |
|  |  | meta_1254 | Unmapped | 2.20 | 1.67 | up | 0 |
|  |  | meta_1256 | Unmapped | 3.25 | 1.66 | up | 0 |
|  |  | meta_1271 | Unmapped | 2.08 | 1.57 | up | 0 |
|  |  | meta_1290 | Unmapped | 2.32 | 1.65 | up | 0 |

­
